# Efficient representation of uncertainty in multiple sequence alignments using directed acyclic graphs

**DOI:** 10.1186/s12859-015-0516-1

**Published:** 2015-04-01

**Authors:** Joseph L Herman, Ádám Novák, Rune Lyngsø, Adrienn Szabó, István Miklós, Jotun Hein

**Affiliations:** 1 Department of Statistics, University of Oxford, 1 South Parks Road, Oxford, OX1 3TG UK; 2 Division of Mathematical Biology, National Institute of Medical Research,, The Ridgeway, London, NW7 1AA UK; 3 Institute of Computer Science and Control, Hungarian Academy of Sciences, Lagymanyosi u. 11., Budapest, 1111 Hungary; 4 Department of Stochastics, Rényi Institute, Reáltanoda u. 13-15, Budapest, 1053 Hungary

**Keywords:** Alignment graphs, Statistical alignment, Alignment uncertainty, Multiple sequence alignment

## Abstract

**Background:**

A standard procedure in many areas of bioinformatics is to use a single multiple sequence alignment (MSA) as the basis for various types of analysis. However, downstream results may be highly sensitive to the alignment used, and neglecting the uncertainty in the alignment can lead to significant bias in the resulting inference. In recent years, a number of approaches have been developed for probabilistic sampling of alignments, rather than simply generating a single optimum. However, this type of probabilistic information is currently not widely used in the context of downstream inference, since most existing algorithms are set up to make use of a single alignment.

**Results:**

In this work we present a framework for representing a set of sampled alignments as a directed acyclic graph (DAG) whose nodes are alignment columns; each path through this DAG then represents a valid alignment. Since the probabilities of individual columns can be estimated from empirical frequencies, this approach enables sample-based estimation of posterior alignment probabilities. Moreover, due to conditional independencies between columns, the graph structure encodes a much larger set of alignments than the original set of sampled MSAs, such that the effective sample size is greatly increased.

**Conclusions:**

The alignment DAG provides a natural way to represent a distribution in the space of MSAs, and allows for existing algorithms to be efficiently scaled up to operate on large sets of alignments. As an example, we show how this can be used to compute marginal probabilities for tree topologies, averaging over a very large number of MSAs. This framework can also be used to generate a statistically meaningful summary alignment; example applications show that this summary alignment is consistently more accurate than the majority of the alignment samples, leading to improvements in downstream tree inference.

Implementations of the methods described in this article are available at http://statalign.github.io/WeaveAlign.

**Electronic supplementary material:**

The online version of this article (doi:10.1186/s12859-015-0516-1) contains supplementary material, which is available to authorized users.

## Background

Sequence alignment is one of the most intensely studied problems in bioinformatics, and is an important step in a wide range of different analyses, including identification of conserved motifs [1], analysis of molecular coevolution [2-4], estimation of phylogenies [5], and homology-based protein structure prediction [6,7].

Many of the most popular alignment methods seek to compute a single optimal alignment, using dynamic programming algorithms [8,9] as well as a variety of heuristic procedures [10-15]. Similar approaches can be used to find maximum likelihood alignments under certain probabilistic models of insertion, deletion and substitution events [16-20].

### Effect of alignment on downstream inference

It has become increasingly clear in recent years that downstream analyses are often highly sensitive to the specific choice of alignment. There may be many plausible but suboptimal alignments within the vicinity of the optimum, containing additional—often complementary—information regarding the evolutionary relationships between the sequences [21]; selecting a single point estimate results in the loss of this additional information, and fails to account for the statistical uncertainty associated with different regions of the alignment [22].

A number of studies have highlighted the impact of the choice of alignment on subsequent phylogenetic inference [23-31]; in many cases different alignment methods, or different guide trees, can give rise to very different phylogenies [23,32-36]. Sensitivity to the alignment is also observed in the context of many other types of downstream analysis, including homology modelling of protein structures [37-39], detection of correlated evolution [40,41], prediction of RNA secondary structure [42], and inference of positive selection [36,43-45].

### Filtering methods

A common approach to tackling the issue of alignment uncertainty has been to attempt to annotate particular regions of the alignment as unreliable, and to remove these before carrying out subsequent analysis. Filtering methods have in some cases been observed to yield improved inference for phylogenies [46-48] and positive selection [44,45].

However, the specific choice of filtering method may have a strong influence on the results [49], and uncertain regions of the alignment may also contain important information that is lost through the use of such methods. For example, tree accuracy is not related in a straightforward fashion to alignment uncertainty [27], and seemingly unreliable regions may be important for accurately resolving phylogenies [50,51]. Regions of high alignment uncertainty can also correspond to sites with higher indel rates [22,52], as well as regions of structural variability [53] or intrinsic disorder [54] in protein structures, and filtering these out may lead to unpredictable biases in subsequent analysis.

### Joint sampling approaches

Within the Bayesian paradigm, alignment uncertainty can be addressed in a more methodical fashion by considering alignments, along with other parameters of interest, as samples from an unknown *posterior* distribution. In this framework, regions of high alignment variability then correspond to regions of high variance in the posterior. The last decade has seen the development of several fully Bayesian approaches for performing joint inference on alignments along with other objects of interest, such as mutation rates [55], phylogenetic trees [56-58], information about the evolution of protein structure [59-62], and the locations of putative regulatory elements [63-65]; inference on these quantities after accounting for alignment uncertainty can then be obtained by averaging over alignments according to their posterior probability under the joint model.

However, although such approaches may be analytically tractable for comparison of a small number of sequences [63,64,66], the computational complexity involved in analysing these hierarchical joint models typically does not scale well with the number of sequences; procedures such as Markov chain Monte Carlo can only increase the range of tractability to a limited extent [56,57,65]. Moreover, adding in another level of annotation or information may require a new model to be formulated, such that in many cases this fully Bayesian approach may be impractical for problems of interest.

### Alternatives to joint sampling

In this work we focus on a tractable alternative that can be used when joint sampling approaches are impractical. This approach takes a collection of alignments sampled according to a particular model, and uses an efficient graph-based representation to generate a much larger set of possible alignments from the initial collection. The acyclic structure of the graph allows many types of analysis to be easily carried out on the whole ensemble of alignments rather than just a single representative, such that the alignment uncertainty quantified by the ensemble can be incorporated into downstream analysis without the need for designing computationally intensive joint sampling approaches. If a single representative of the ensemble is required, this framework also allows for the efficient computation of the single alignment that maximises the expected value of a variety of different accuracy scores.

The simple and computationally efficient nature of this representation makes it practical to adopt a more principled, probabilistic approach to quantifying and making use of alignment uncertainty, and we discuss examples of cases where this may prove particularly useful.

## Quantifying alignment uncertainty

A number of different approaches have been developed for quantifying the uncertainty associated with a multiple sequence alignment. Many of these methods focus on the notion of alignment *reliability*, i.e. the degree to which a particular alignment (or regions thereof) can be trusted as a prediction of the homology between the sequences.

One set of approaches involves computing scores or summary statistics on a single alignment of interest, using these as a measure of reliability of the alignment. Some of these approaches equate reliability of a particular alignment column with a high score under the model used to generate the alignment [67], the justification being that low-scoring columns are harder to distinguish from random noise, and so are more likely to contain erroneous homology statements; others generate the alignment using one scoring scheme, and measure its ‘reasonableness’ based upon another set of criteria [68,69], which may involve looking at the deviation of summary statistics from their expected background distribution under the null hypothesis of no homology [70,71]. One potential issue with some of these approaches is that they introduce a bias towards highly conserved regions, since they do not distinguish between evolutionary variability and statistical uncertainty, often using the term *alignment quality* as a synonym for reliability.

An alternative approach, first mentioned by [49], involves generating a set of plausible alignments, and assessing the alignment uncertainty by measuring the similarity between the alignments in this set. This type of *consistency*- or *congruence*-based approach has a more natural statistical interpretation, but requires a method of generating alternative alignments, as well as a measure of alignment similarity or distance; the interpretation of the resulting measures of uncertainty may depend heavily on these two factors.

### Generating sets of alignments

A variety of heuristic methods have been developed in order to generate sets of alignments for the purposes of measuring uncertainty. Perhaps the simplest of these is to align the same sequences with the residue order reversed [72], although the efficacy of this technique is questionable [73,74]. Another class of methods generates alternative alignments by perturbing parameters such as the guide tree [75,76], gap opening and extension penalties [77,78], and substitution matrices [79,80], and recomputing the optimal alignment with these alternative parameters. However, in all these cases the types of perturbations applied to the parameters will affect the resulting estimates of uncertainty in an unpredictable fashion [70].

Another approach is to look at a set of suboptimal alignments under a particular scoring scheme, given fixed parameters [81-83], using these to search for regions of consistency [84-86]. The variability among these suboptimal alignments can then be converted into a measure of statistical uncertainty, using an approximation to the distribution of scores, for example using an extreme value distribution [87].

### A Bayesian approach

Within a Bayesian framework, the collection of plausible alignments can be identified with the *posterior distribution* of the alignment given the sequences and other model parameters; this leads to a probabilistic interpretation of alignment uncertainty, whereby the fraction of alignments containing a particular homology statement is a measure of the posterior probability of that homology statement.

For the pairwise case, alignments can often be sampled exactly from their posterior distribution under a particular evolutionary model using a dynamic programming approach [88-90]. However, for multiple sequences such approaches rapidly become computationally infeasible, and other types of procedures must be used. A popular option is to use Markov chain Monte Carlo (MCMC) in order to sample from the posterior distribution of alignments [55-58,60,61,65,91-94]. The main advantage of the MCMC approach is that it is guaranteed to sample alignments from the correct probability distribution, provided that the simulation is run for long enough to ensure convergence, although this may require significant amounts of runtime.

## Representing the distribution of sampled alignments

Once a set of plausible alignments has been generated, a common issue that arises is how to represent and/or summarise this set in a useful fashion. In a Bayesian context this entails representing the approximation to the posterior distribution over alignments, given a collection of samples. We shall present here a graph-based formulation that allows for a compact representation of this distribution, permitting algorithms to be designed for efficient inference on exponentially large sets of alignments derived from a collection of samples.

### Mapping columns to dynamic programming tables

A multiple sequence alignment can be represented as a path through a multidimensional matrix; an edge from one cell of the matrix to an adjacent cell represents a particular set of homology statements, synonymous with a column in the alignment. It is a straightforward extension to consider a *set* of alignments as a set of paths in such a matrix [95].

To formalise this intuition, we introduce a bijection between the set of alignment columns and the set of edges connecting cells in the multidimensional dynamic programming matrix, based on the coding scheme described in the supplementary section of Satija *et al.* [65]. More specifically, a column *X* containing *N* rows can be mapped to an *N*-tuple *C*(*X*)=(*c*(*X*_1_),…,*c*(*X*_*N*_)), where *c*(*X*_*i*_) is defined as

(1) $$ c(X_{i}) = \left\{ \begin{array}{cl} 2j-1 & \text{if}\,\, X_{i} = s^{(i)}_{j}\\ 2j & \text{if}\,\, X_{i} = \text{gap}, \text{between}\,\, s^{(i)}_{j} \text{and}\,\, s^{(i)}_{j+1} \end{array} \right.  $$

where $s^{(i)}_{j}$ is the *j*th character of the *i*th sequence, such that *C*(*X*) corresponds to the coordinates of the midpoint of an edge connecting two cells in the matrix. We will also introduce initial and terminal columns, *X* ^(0)^ and *X* ^(*T*)^, which can be thought of as all-gap columns preceding the first characters and following the last characters of the sequences, respectively. These will therefore be encoded as *C*(*X* ^(0)^)=(0,…,0) and *C*(*X* ^(*T*)^)=(2*L*_1_,…,2*L*_*m*_) where *L*_*i*_ is the length of the *i* ^*th*^ sequence.

**Figure 1:**
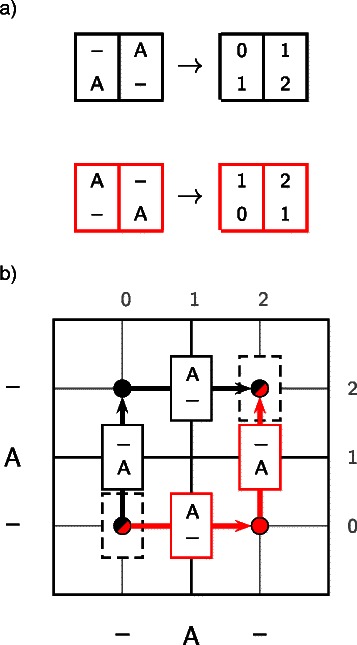
Correspondence between alignment columns and edges connecting cells in a dynamic programming matrix, illustrated for pairwise alignment. In order to permit a directed acyclic graph representation of the space of possible alignments, each column is given a code that distinguishes between gaps based upon where they occur in the alignment. The coding for each column for the two alignments shown in panel ***a)*** represents a bijection to the midpoints of edges connecting cells in the dynamic programming table in panel ***b)***. Cell boundaries are indicated by thicker gridlines, and the finer gridlines indicate the column coding corresponding to each position, as labelled on the top and right axes. These codings are derived from the characters shown on the bottom and left axes. The midpoint of each cell is labelled with a circle, and each edge is annotated with a rectangle denoting the corresponding column. Each path from *X* ^(0)^ to *X* ^(*T*)^ (shown as dashed columns at (0,0) and (2,2), respectively) represents a valid alignment.

It is then possible to map any global alignment, *A*, to a path, *C*(*A*)=(*X* ^(0)^,*C*(*A* ^(1)^),…,*C*(*A* ^(*L*)^),*X* ^(*T*)^) through the dynamic programming matrix (*see Figure* 1).

### Intersections between alignments

The paths corresponding to a particular set of alignments may intersect at one or more points in the matrix; as first discussed by Bucka-Lassen *et al.* [95], subpaths can be ‘spliced’ at these points in order to generate new alignments. This approach was originally used to create an augmented search space for locating an optimal alignment [95,96], and more recently has been used as part of a progressive alignment algorithm that keeps track of suboptimal alignments [97].

**Figure 2:**
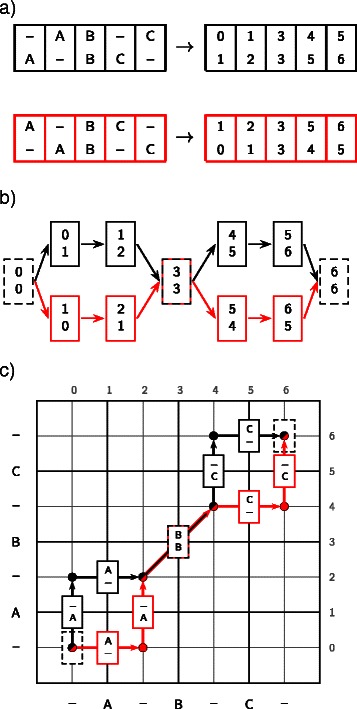
Interchanges between alignments can result in a multiplication of the number of possible paths through the DAG.***a)*** Two alignments coded under the map *C*, as described in Equation (1). ***b)*** The resulting alignment DAG contains an interchange column, such that there are four paths through the DAG, arising from only two alignments. ***c)*** Correspondence between alignment columns and edges connecting cells in a dynamic programming matrix.

**Figure 3:**
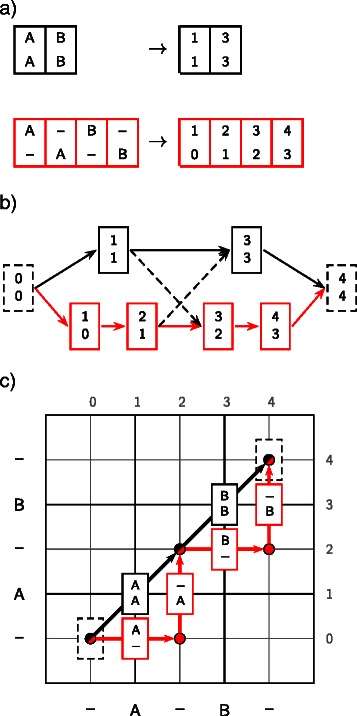
Crossovers between two alignments containing no interchange columns.***a)*** Two alignments coded under the map *C*, as described in Equation (1). ***b)*** The resulting alignment DAG allows for crossovers between these alignments, such that there are four possible paths through the DAG, two of which include pairs of columns that are not observed in the input alignments (dashed lines). ***c)*** Correspondence between alignment columns and edges connecting cells in a dynamic programming matrix.

The types of intersections fall into two categories, as illustrated in Figures 2 and 3. The first of these, which we term an *interchange*, results when two or more sampled alignments contain the same column, but with a different predecessor and successor, as shown in Figure 2. The second type of intersection is termed a *crossover*, whereby two or more sampled alignments contain pairs of *equivalent* columns, as shown in Figure 3. Each interchange or crossover can result in a multiplication of the number of possible ways of recombining the sampled alignments, such that the total number of alignments is greatly increased.

As a result of this, an initial set of alignments sampled according to a particular model can be used to generate a much larger set of alignments sampled according to the same distribution, as we shall examine in further detail in the subsequent section.

### Equivalence classes of columns

**Figure 4:**
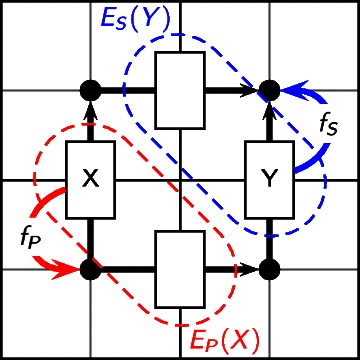
Predecessor and successor functions, and equivalence classes of columns. The predecessor and successor functions (*f*_*P*_ and *f*_*S*_ respectively) map from columns (edges) to nodes (circles) in the dynamic programming matrix. All columns mapping to a particular node under *f*_*P*_ share the same set of possible predecessor columns, and are grouped together in an equivalence class, denoted by *E*_*P*_ (shown in red). An analogous definition holds for *E*_*S*_ (blue).

In order to delineate the ways in which a set of columns can be recombined to form new alignments, we introduce the *predecessor* and *successor* functions, *f*_*P*_ and *f*_*S*_ respectively. The functions *f*_*P*_ and *f*_*S*_ take the coordinates of a column *X* as input, and return the coordinates of an equivalence class of columns, corresponding to the midpoint of the predecessor (respectively successor) cell in the multidimensional matrix. Each column mapping to a particular *f*_*P*_- or *f*_*S*_-equivalence class can follow the same set of predecessor or successor columns, respectively (*see Figure* 4).

Denoting the *i*th coordinate of the output by *f*_*P*_(*X*)_*i*_ and *f*_*S*_(*X*)_*i*_, the functions are defined such that

(2) $$\begin{array}{*{20}l} f_{P}(X)_{i} &= c(X_{i}) - c(X_{i}) \, \text{mod}\, 2 \end{array} $$

(3) $$\begin{array}{*{20}l}  f_{S}(X)_{i} &= c(X_{i}) + c(X_{i}) \, \text{mod}\, 2 \end{array} $$

The original column coding is then uniquely recovered by the backwards mapping

(4) $$ C(X) = (f_{P}(X) + f_{S}(X))/2   $$

The equivalence class *E*_*P*_(*X*) is then defined as the set of columns, {*X* ^′^∣*f*_*P*_(*X* ^′^)=*f*_*P*_(*X*)}, with *E*_*S*_(*X*) similarly defined.

Using the definitions above, a column *X* ^′^ is a predecessor of *X* if and only if *f*_*S*_(*X* ^′^)=*f*_*P*_(*X*), since any path connecting them must pass through the separating equivalence class *E*_*S*_(*X* ^′^)≡*E*_*P*_(*X*). We will use the notation $\mathcal {P}(X) \equiv \{ X^{\prime } \mid f_{S}(X^{\prime }) = f_{P}(X) \}$ to denote the set of predecessors of *X*.

### The alignment column graph

We can then define the *alignment column graph*, $\mathcal {D}(\Xi)$, of a set of columns, *Ξ*, as a graph whose nodes are the columns in *Ξ*, with a directed edge from column *X* to column *X* ^′^ if and only if *f*_*S*_(*X*)=*f*_*P*_(*X* ^′^), which we write as $X \ltimes X^{\prime }$. From the definitions in Equations (2) and (3), we have *f*_*P*_(*X*)<*f*_*S*_(*X*) for all *X*, in the sense that *f*_*P*_(*X*)_*i*_≤*f*_*S*_(*X*)_*i*_ for all *i*, with no column having *f*_*S*_(*X*)=*f*_*P*_(*X*) unless it consists of all gaps. This ensures that the alignment column graph is acyclic, since it is never possible to return to the same equivalence class by following a set of directed edges in the graph.

**Figure 5:**
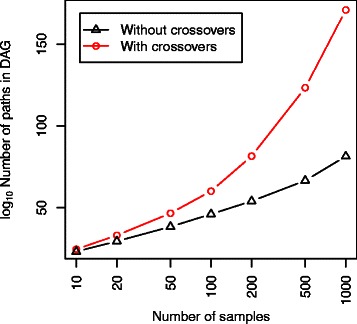
The number of paths through the alignment column graph as a function of the number of alignments used to generate the graph. Shown for a set of 10 sequences simulated using DAWG (simulation procedure described in the main text). When crossovers are allowed (corresponding to a mean-field approximation for the conditional marginal for each column), the number of paths increases super-exponentially, resulting in a much higher coverage of the space of possible alignments, and hence more accurate approximations to the posterior probability for each path (*see Figure* 8).

Each directed path through the column graph generates a valid alignment; a *global alignment* is a valid alignment that begins at *X* ^(0)^ and ends at *X* ^(*T*)^, such that the number of possible global alignments is equal to the number of distinct paths in $\mathcal {D}(\Xi)$ that lead from *X* ^(0)^ to *X* ^(*T*)^. This is typically very large, growing rapidly with the number of intersection points between the alignments used to generate the graph (*see Figure* 5).

Implicit in the definition of the mapping in Equation (1) is a distinction between gaps based on their position in the alignment, such that the two situations shown in Figure 1 represent distinct alignments, each yielding two different pairs of columns. This assumption is necessary in order to generate a sparse graph; treating all gaps as equivalent is tantamount to replicating each gap-containing column onto all parallels, such that the graph in general becomes maximally dense, making efficient algorithms difficult to implement (*see Additional file* 1 *: Figure S2*).

## Probability distributions on alignment DAGs

Due to the high-dimensional nature of the alignment space, in any particular set each alignment will typically occur with a very low frequency; even the most likely alignment may only be sampled once, if at all [93,98]. As such, the relative probabilities of entire alignments are difficult—if not impossible—to estimate directly by their observed frequencies. However, a particular column may occur in many different alignments, allowing the *marginal* probability of each column, averaged over all alignments, to be estimated much more efficiently [93,99]. As we shall discuss, they also represent useful summary statistics of the full distribution.

### Alignment probabilities in terms of pair marginals

For general evolutionary models, the DAG can be used to construct a factored approximation to the full distribution over alignments; this factored distribution corresponds to a graphical model with dependencies between neighbouring columns defined by the edges in the DAG. Under this factored approximation, the probability of an alignment (corresponding to a path through the DAG) can be written in the form

(5) $$\begin{array}{@{}rcl@{}} &p(A) = p\left(A^{(1)}\right) \prod\limits_{i=2}^{L} p\left(A^{(i)} \mid A^{(i-1)}\right)  \end{array} $$

where

(6) $$\begin{array}{@{}rcl@{}} &p\left(A^{(i)} \mid A^{(i-1)}\right) = p\left(A^{(i)},A^{(i-1)}\right) / p\left(A^{(i-1)}\right). \end{array} $$

For evolutionary models based on first-order hidden Markov models (HMMs) (such as the one shown in Additional file 1: Figure S4), the pair-marginal representation is exact, since the dependencies in the model are equivalent to those in the DAG. For models with non-local dependencies between columns, simply setting the pair marginals to be equal to the observed pair marginals minimises the Kullback-Liebler divergence from the full empirical distribution to the pair-marginal approximation (*see Additional file* 1 *: Section S4*).

### Motivations for using factored approximations

There are three main reasons for making use of factored approximations of this type:

- The number of possible column pairs is many orders of magnitude lower than the number of alignments, such that pair marginals can be estimated much more reliably from observed frequencies. These can then be used to construct more accurate estimates of the overall joint probability.
- Expression of the joint in terms of pair-marginals allows for interchanges in the alignment DAG (*cf. Figure* 2), allowing many alternative alignments to be generated from an initial collection of samples.
- Factorisation of the probability into a product of local terms allows for efficient algorithms to be implemented on the DAG structure.

We discuss these factors in further detail below.

### Mean-field approximation

As well as distributions involving pair terms, we will also consider a *mean-field* type approximation, whereby the conditional distribution of each column is given a specific predecessor [cf. Equation (6)] is replaced by an average over all predecessors:

(7) $$\begin{array}{@{}rcl@{}} p(X \mid \mathcal{P}(X)) &=& p(X,\mathcal{P}(X)) / p(\mathcal{P}(X)) \end{array} $$

(8) $$\begin{array}{@{}rcl@{}}  &=& p(X) / \sum\limits_{X^{\prime} \ltimes X} p(X^{\prime}) \end{array} $$

where $p(X \mid \mathcal {P}(X))$ is the probability of column *X* given that any one of its possible predecessors is in the alignment. The second line uses the identities $p(X, \mathcal {P}(X)) \equiv p(X)$ (since a column can only be present if one of its predecessors is present), and $p(\mathcal {P}(X)) \equiv \sum _{X^{\prime }\ltimes X} p(X)$ (since only one member of an equivalence class can be present in any particular alignment).

An important corollary of the expression in Equation (8) is that single-column marginals are sufficient to reconstruct the mean-field approximation to the joint probability; this has several important consequences, as we shall discuss below.

### Motivations for using the mean-field approximation

The mean-field approximation described above is exact for fully independent sites models, for example pair HMMs with non-affine models for indels. For more general HMMs, there are three major advantages associated with using this approximation rather than the pair-marginal formulation:

- Since the number of possible columns is substantially less than the number of possible column pairs, it is easier to obtain reliable estimates of single-column marginals from a collection of alignment samples. Hence, the mean-field approximation is likely to be more accurate for lower sample sizes.
- The use of single-column marginals allows for crossovers in the alignment DAG (*cf. Figure* 3), whereas the pair-marginal expression will assign a weight of zero to any pairs that are not observed, hence only permitting interchanges of the form shown in Figure 2. This allows for a higher effective sample size for the alignments under the mean-field approximation, with more alternative alignments generated from the same collection of samples.
- Restricting to single-column marginals more efficient algorithms to be constructed, involving one-step rather than two-step recursions.

In the rest of this section, we examine these points in further detail.

### Estimating marginal probabilities

For a pairwise alignment, column marginals can be easily represented using a matrix in which the (*i*,*j*) entry contains the marginal probability $p(s^{(1)}_{i} \diamond s^{(2)}_{j})$, where $s^{(1)}_{i}$ and $s^{(2)}_{j}$ are the *i*th and *j*th characters in two sequences *s* ^(1)^ and *s* ^(2)^, and the symbol ◇ denotes homology. When only two sequences are under comparison, dynamic programming recursions allow for the exact computation of these marginal probabilities under certain types of evolutionary models [55,100,101].

In the multiple sequence case, such exact computations are typically infeasible. However, if we are provided with a set, , of sampled alignments, an estimate of the marginal probability of each column (after coding) can be computed as the proportion of the alignments in  that contain the column, weighted according to the alignment probability. This can be written using the following indicator function notation
(9)


If we consider a *multiset*, $\mathcal {A}^{+}$, containing global alignments sampled one or more times according to their probability, then the factor *p*(*A*) can be replaced by the relative frequencies of the sampled alignments. The estimator for the marginal probability $\hat {p}_{C}(X)$ is then proportional to the fraction of sampled alignments containing a column *X* ^′^ for which *C*(*X* ^′^)=*C*(*X*):

(10) $$\begin{array}{@{}rcl@{}} \hat{p}_{C}(X) &= n_{C}(X,\mathcal{A}^{+}) /|\mathcal{A}^{+}| \end{array} $$

with $n_{C}(X,\mathcal {A}^{+})$ denoting the number of occurrences of *C*(*X*) across all the alignments contained in the multiset $\mathcal {A}^{+}$. If enough alignments are sampled from the correct distribution, the above estimator will converge to the true value *p*_*C*_(*X*). Although conditional marginals can also be computed from local alignments (*see Additional file* 1 *: Section S1*), in this work we will consider only global alignments, in the interests of simplicity.

Since in most cases each sampled alignment will be unique, due to the high dimensional nature of the state space, in the rest of this manuscript we will refer only to the set  rather than the multiset $\mathcal {A}^{+}$. However, for cases where uncertainty is low, and the same alignment may be sampled more than once, it is important to treat each replica as an independent sample when computing marginal probabilities.

Marginal probabilities can also be estimated for pairs of columns using observed pair frequencies. However, the space of possible pairs of columns can be much larger than the space of columns; in the worst case this will be by a factor of $\mathcal {O}(2^{N})$, where *N* is the number of sequences, since this is the maximum size of an equivalence class. Hence, a larger number of alignment samples will be needed to obtain accurate estimates for pair marginals. As we shall see, this means that pair-based reconstructions of joint probabilities are typically less accurate unless a very large number of samples is used.

### Reconstructing alignment probabilities from marginals

Generally, with sampling-based procedures such as MCMC, posterior probabilities are estimated via sampled frequencies. However, in the case of a very high dimensional parameter such as a multiple sequence alignment, each point in the space may only be visited once, such that it is not possible to estimate posterior probabilities based on these frequencies.

As discussed above, the set of marginal probabilities for each column (or pair of neighbouring columns) can be used to reconstruct the posterior probability for any particular alignment, via Equation (5). Although the likelihood for each sampled alignment will often be known as a by-product of the sampling procedure, the *marginal* posterior probability of each alignment after integrating over other unknown parameters (for example indel rates), will typically not be known. Hence, the DAG-based approach presented here represents a useful way to calculate posterior probabilities in such cases. A similar approach has been used recently to compute the posterior probabilities of phylogenetic trees based on the probabilities of each of the constituent clades, under the assumption of conditional independence between clades [102].

**Figure 6:**
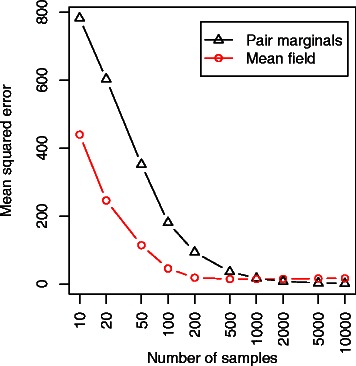
Mean squared error in the approximation to the true posterior, as a function of the number of alignment samples. Shown for the pairwise globin example. Although the pair-HMM involves neighbour-dependent terms (leading to an affine gap penalty), the mean-field approximation leads to a better estimate of the true posterior until around 1000-2000 samples are taken. This is due to the presence of intersections between paths in the alignment DAG, which allows for a higher effective sample size to be obtained from the same number of alignments.

**Figure 7:**
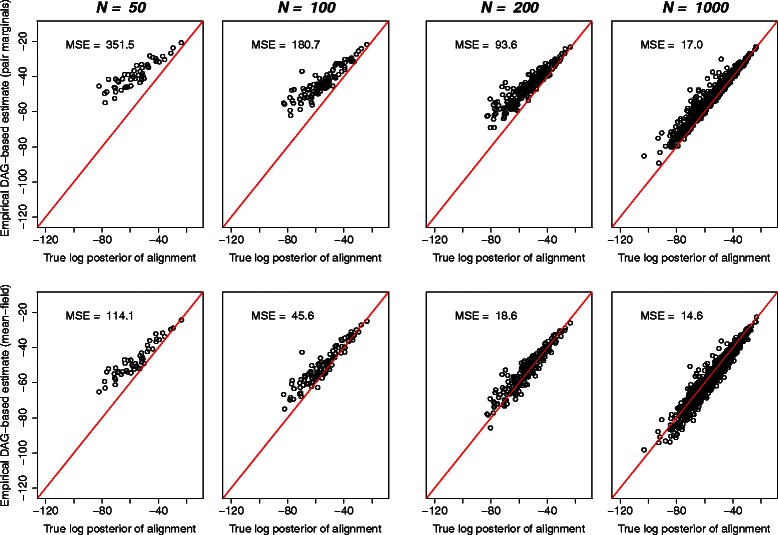
As more alignment samples are taken, the DAG-based estimate of the log posterior probability for each alignment converges towards the true value. The DAG-based probabilities already yield a good estimate when the number of alignments, *N*, is just 100. Shown on the top row are the reconstructed probabilities derived using pair marginals, and on the bottom using the mean field approximation, with the line *y*=*x* overlaid in red. Since each sampled alignment is generally observed only once, the posterior probability estimated directly from alignment frequency would be 1/*N* in each case above. The DAG methodology therefore offers a clear advantage for the purposes of computing posterior alignment probabilities. The mean-field approximation results in a lower mean-squared error (MSE), due to the higher effective sample size (*see Figure* 6).

As an illustration of this procedure, a set of pairwise alignments were sampled from the pair-HMM in Additional file 1: Figure S4, combined with the Dayhoff amino acid rate matrix [103], for two globin sequences (sampled alignments illustrated in Additional file 1: Figure S3). As shown in Figures 6 and 7, the DAG-based estimates of the posterior probability converge towards the true probability as the number of samples is increased, reaching a good agreement after just 200 samples, as measured by the mean-squared error of the logarithm:

(11) $$ MSE(\hat{p}\, ||\, p) = \frac{1}{|\mathcal{A}|}\sum\limits_{A \in \mathcal{A}} (\log \hat{p}(A) - \log p(A))^{2}  $$

For lower numbers of samples, the estimates are more accurate for the more probable alignments, since the more extreme regions of the space are sampled with lower probability, and hence converge more slowly.

Although both pair-marginal and mean-field estimates converge in this case at a similar rate, closer analysis shows that the mean squared error in the approximation to the true posterior is considerably less for the mean-field approximation. This suggests that the improvement obtained by summing over a larger number of paths (*see Figure* 5) outweighs the approximation introduced by averaging over predecessor states, although eventually at around 2000 samples the pair-marginal estimates begin to dominate the mean-field approximation (*see Figure* 6), since the true pair-HMM involves neighbour-dependent terms. The precise location of this crossover point will depend on the degree of neighbour dependency; for a completely site-independent model (e.g. the pair-HMM in Additional file 1: Figure S4 with *δ*=*ε*=*σ*), the single-column marginal estimate always dominates (*see Additional file* 1 *: Figure S7*).

**Figure 8:**
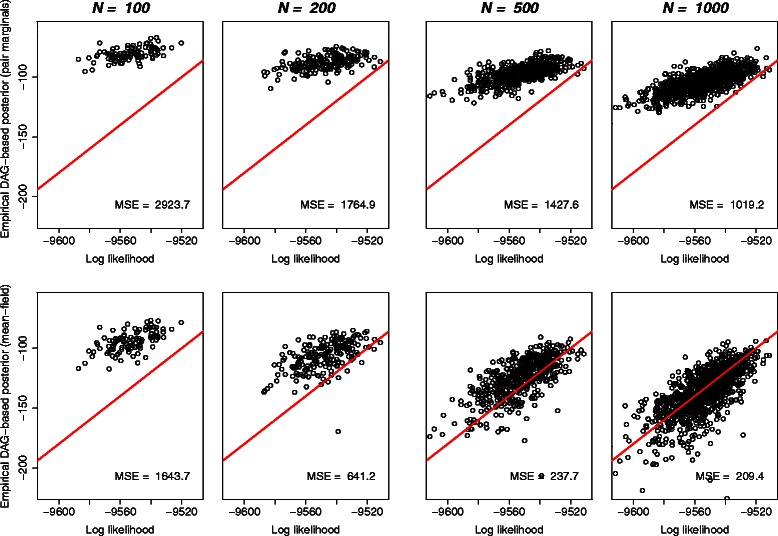
For a larger multiple sequence alignment, the mean-field approximation to the log posterior (bottom row) converges much more quickly than the pair marginal estimate, despite the fact that the indel model used includes neighbour-dependent terms. This is due to the fact that column marginals can be estimated more reliably than pair marginals, combined with the fact that allowing crossovers in the DAG results in a higher effective sample size (*see Figure* 5). Results shown for the simulated dataset described later in the main text, using the TKF92 indel model [17]. In this case the true posterior probability cannot be computed analytically, but the log likelihood (conditional on specific values of the other unknown parameters) is known. Since the log likelihood is expected to be linearly related to the log posterior, convergence can be gauged approximately by assessing the fit to a relationship of *y*=*x*+*k* (overlaid in red, with *k*, the approximate normalising constant, chosen to match the distribution to which the mean-field approximation converges, here *k*=−9420).

This same pattern is observed in a more striking fashion for a larger, 10-sequence alignment, as shown in Figure 8. Moreover, since the space of possible alignments increases very rapidly with the number of sequences, the benefit of using the mean-field approach to boost the effective sample size is greater in the multiple-sequence case, resulting in much faster convergence of the posterior estimates (*see Figure* 8).

### Approximate summation over all alignments

As well as computing the probability of individual paths in the DAG, it is possible to sum over all alignments contained within the DAG using a standard dynamic programming algorithm (*see Additional file* 1 *: Section S5*).

In the pairwise case, where it is possible to analytically compute the sum over all alignments (by filling out the full dynamic programming table), it is possible to examine how much of the posterior mass is contained within the DAG resulting from a particular set of samples.

**Figure 9:**
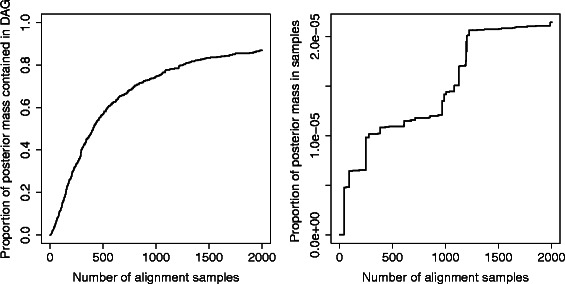
The proportion of the posterior mass contained in paths through the DAG increases rapidly with the number of samples. For the pairwise example discussed in the text, the proportion reaches in the order of 10- 15*%* of the total posterior mass with just 100 samples, increasing to over 80*%* after including 2000 samples (left panel). In contrast, the proportion of posterior mass contained within the individual samples is very small (right panel).

While the probability mass contained within the individual samples increases relatively slowly, and encapsulates only a very small fraction of the total, the proportion of the posterior mass encapsulated in the set of paths through the alignment DAG increases much more rapidly; the DAG contains in the order of 10- 15*%* of the total posterior mass over the entire set of possible alignments with just 100 samples, increasing to around 80*%* after including 2000 samples (*see Figure* 9 *and Additional file* 1 *: Figure S1*).

A similar dynamic programming algorithm can be used to calculate the total number of paths (i.e. alignments) contained within the DAG. Examining the number of paths in the DAG as a function of the number of alignment samples shows a super-exponential relationship when crossovers are allowed, whereas restricting to observed column pairings increases close to exponentially (*see Figure* 5). In the pairwise case, the theoretical maximum can be computed analytically; for the pairwise example discussed above, the total number of paths in the DAG has an upper bound in the order of 10^113^.

## Summarising the alignment distribution

Although the set of alignments encoded by the DAG contains a great deal of additional information beyond that contained in any one alignment, there may be situations where a single alignment is desired as a summary of the distribution. Due to the high-dimensional and constrained nature of the state space, standard summary statistics such as the mean are not applicable in this case [104].

### Finding the MAP alignment

One of the simplest summaries of the distribution is the *maximum a posteriori* (MAP) alignment. As mentioned earlier, estimation of this quantity directly from sample frequencies is typically very unreliable, since each alignment is typically only sampled once, such that each sample has the same empirical posterior probability. However, as discussed above, the DAG-based approach to estimating posterior probabilities can be used to obtain good estimates of the probability for each possible alignment contained in the DAG. We can then use the fact that the DAG-based log posterior is additive over the columns in the alignment

(12) $$\begin{array}{@{}rcl@{}} & \log p(A) = \log p\left(A^{(1)}\right) + \sum\limits_{i=2}^{L} \log p\left(A^{(i)} \mid A^{(i-1)}\right) \end{array} $$

such that the path with the maximum posterior can be found using standard dynamic programming algorithms for DAGs (*see Algorithm 1*).



Nevertheless, due to large size of the space of possible alignments, there may be a large number of very similar alignments with very similar posterior probability. Hence, quantities such as the MAP can be poor summary statistics of the distribution [58,93,94]. Instead, we will consider alternative types of summary alignments that account for the uncertainty contained within the DAG.

### Loss function formulation

The problem of choosing a single summary alignment can be approached within a decision theoretical framework, whereby the choice of summary is designed to minimise the expected value of a particular loss function, also known as the *posterior risk* [104]. For a loss function defined in terms of alignment *accuracy*, minimising the posterior risk is equivalent to selecting the *maximum expected accuracy* alignment [98,105,106].

The *loss* of an alignment, *A*, with respect to a reference alignment, *A* ^′^, will be denoted by *L*(*A* || *A* ^′^), and represents a penalty associated with choosing alignment *A*, given that the true alignment is *A* ^′^. The posterior risk associated with *A* can then be defined as

(13) $$\begin{array}{@{}rcl@{}} \mathcal{R}(A) &=& \mathbb{E}\left [ \mathcal{L}(A\ ||\ A^{\prime}) \right ] \end{array} $$

(14) $$\begin{array}{@{}rcl@{}} &=& \sum\limits_{A^{\prime}} p(A^{\prime}) \mathcal{L}(A\ ||\ A^{\prime}) \end{array} $$

where the sum over *A* ^′^ includes all alignments. The minimum-risk alignment is then $\hat {A} = \arg \min _{A} \mathcal {R}(A)$.

For loss functions defined as a sum over columns (equivalent to the *pointwise gain* functions discussed by Hamada *et al.* [106]), we have

(15) $$\begin{array}{@{}rcl@{}} \mathcal{L}(A\, ||\, A^{\prime}) &= k \sum\limits_{X \in A} \mathcal{L}(X\, ||\, A^{\prime})  \end{array} $$

where *k* is independent of *A*. In order to define the loss for a particular column, we will consider the following four categories of columns in the predicted alignment, *A*:

- True positives (TP) = Columns correctly present
- False positives (FP) = Columns incorrectly present
- True negatives (TN) = Columns correctly absent
- False negatives (FN) = Columns incorrectly absent

such that *T* *P*∪*F* *P*∪*T* *N*∪*F* *N*=*Ξ*, the set of all observed columns.

Generally we will not be interested in the number of negatives (i.e. columns not included in the alignment), since this will depend on how many alignment samples are used to generate the DAG. We will therefore focus on loss functions of the form
(16)



(17)


where *f* is a bijective function operating on columns, with *f*(*A*)=(*f*(*A* ^(1)^,…,*f*(*A* ^(*L*)^)), and *λ*_*FP*_ and *ρ*_*TP*_ are loss/reward functions associated with false positives and true positives respectively.

As shown in Additional file 1: Section S2, the posterior risk can then be written as

(18) $$\begin{array}{@{}rcl@{}} \mathcal{R}_{f}(A) &\propto \sum\limits_{j=1}^{L_{A}} \left[g - p_{f}(A^{(j)})\right]  \end{array} $$

where  is the marginal probability of column *X* being present according to the mapping specified by *f*, and *g*=*λ*_*FP*_/(*ρ*_*TP*_+*λ*_*FP*_) is penalty term that penalises longer alignments by a factor proportional to the penalty on false positives. In contrast to an arbitrarily chosen gap penalty, the penalty, *g*, has a direct interpretation in this case. It is also a straightforward extension to allow *λ*_*FP*_ and *ρ*_*TP*_, and hence *g*, to depend on the specific column, *X*, for example penalising a false positive proportionally to the number of non-gap characters contained in the column.

### Loss functions corresponding to common accuracy measures

The simplest choice in Equation (16) is to set *f*(*X*)=*C*(*X*) as defined in Equation (1), such that *p*_*f*_(*X*) is equal to the marginal probability as defined in Equation (9). The loss function formulation can also be used to represent commonly used measures of *alignment accuracy*. Perhaps the simplest of these is the so-called *column score*; this measures the proportion of correct columns, but without differentiating between the positions of the gaps. This can be defined more formally by first introducing an alternative column mapping, *C* ^+^(*X*)=(*c* ^+^(*X*_1_),…,*c* ^+^(*X*_*N*_)), which groups together all columns that contain the same non-gap characters:

(19) $$ c^{+}(X_{i}) = \left\{ \begin{array}{cl} 2j-1 & \text{if}\,\, X_{i} = s^{(i)}_{j}\\ 0 & \text{if}\,\, X_{i} = \text{gap} \end{array} \right.  $$

**Figure 10:**
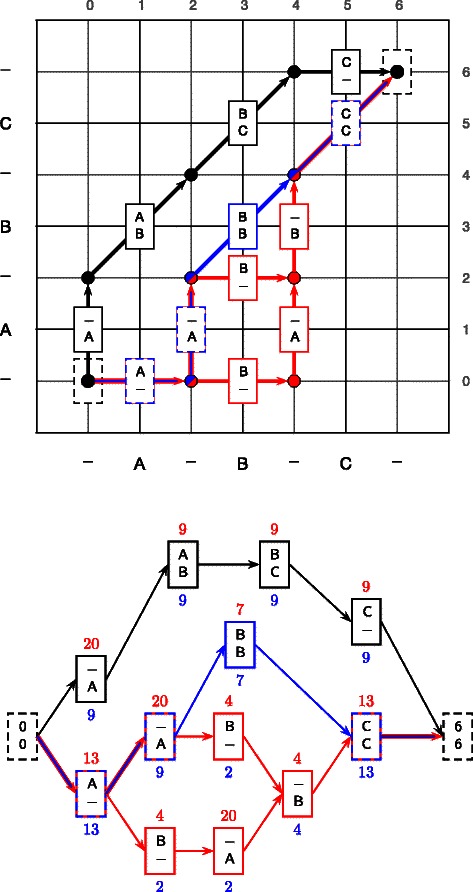
The minimum-risk path under the *C*-based loss function (blue) may not be the same as that under the *C* ^+^-based loss function (red). Column frequencies are shown in blue below each column, and the *p*_*C*+_ marginals shown in red above (as frequencies from a total of 20 samples). In this case, there are two equivalent paths with the same *C* ^+^-score.

The column score for an alignment, *A*, with respect to a reference, *A* ^′^, can then be defined as $-\mathcal {L}_{C^{+}}(A\ ||\ A^{\prime })$, with *λ*_*FP*_ set to zero. Since we have
(20)


and hence $\phantom {\dot {i}\!}p_{C^{+}}(X) \geq p_{C}(X)$ and $\hat {p}_{C^{+}}(X) \geq \hat {p}_{C}(X)$, the *C* ^+^-risk, i.e. $\mathcal {R}_{C^{+}}$, represents an upper bound to the *C*-risk, $\mathcal {R}_{C}$. As shown in Figure 10, the alignment minimising the *C* ^+^-risk will not in general be the same as the alignment minimising the *C*-risk, although there may be considerable overlap.

As discussed in Additional file 1: Section S3, the above approach can easily be extended to make use of a function, *f*, which splits a column up into a set of pairwise homology statements. This allows various pairwise accuracy scores to be expressed in terms of similar types of loss functions.

### Modeller scores

One other class of loss function worth mentioning here is the so-called *modeller* version of each of the aforementioned scores, $\mathcal {L}^{m}_{f}(A\, ||\, A^{\prime })$, which involve normalising $\mathcal {L}_{f}(A\, ||\, A^{\prime })$ by the length of the predicted alignment, *A*. For example, the modeller *C*-score, corresponding to $\mathcal {L}^{m}_{C}(A\, ||\, A^{\prime })$, was considered by Collingridge and Kelly [79]; as we shall see, the dependence on the length of the predicted alignment precludes the use of exact optimisation algorithms for loss functions such as this.

### Efficient algorithms

In general, minimising the expectation of any of the aforementioned loss functions over the space of all possible multiple alignments is a problem whose complexity grows exponentially with the number of sequences [107]. For the pairwise case, the minimum-risk/maximum expected accuracy problem can be implemented efficiently using standard dynamic programming algorithms [22,60,61,88,94,98,108-110]; for multiple sequences approximate techniques have generally been used, including simulated annealing [20,111,111,112], and greedy [113] or progressive alignment algorithms [105,114-116].

**Figure 11:**
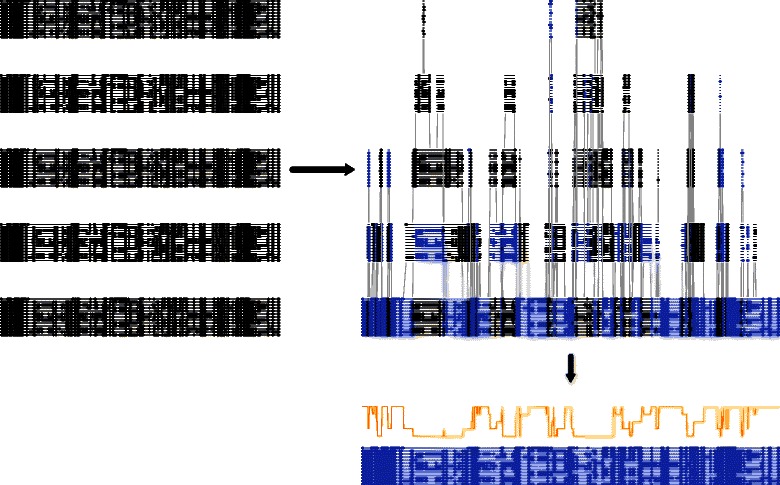
A collection of alignment samples can be combined into a DAG structure, and a summary algorithm generated using efficient algorithms. The graph can be visualised by vertically ordering columns based on the longest path length to the end of the DAG (as shown above). Each path represents a valid combination of the columns in the input alignments, with valid recombinations shown as grey lines in the above figure. The *maximum a posteriori* or minimal-risk path can then be found efficiently using linear-time algorithms, yielding a single summary alignment (shown in blue) that accounts for the uncertainty in the alignment set, and can be annotated with posterior probabilities for each column (shown in orange).



However, if the solution set is restricted to the (still very large) space of alignments encoded in the DAG, any risk function that is additive over columns [in the sense of Equation (15)] can be minimised in time linear in the number of columns in the DAG, by making use of efficient maximum-weight path algorithms (*see Algorithm 2*; Figure 11). This type of approach was first mentioned by Lunter *et al.* [93], and an implementation described by Satija *et al.* [65], although these previous studies did not examine the algorithm in terms of loss functions.

The same approach cannot be applied to minimise the risk under modeller variants, however, since the contribution of each column to the partial sum at each step in the dynamic programming algorithm depends on the unknown final alignment length. Collingridge and Kelly recently presented an algorithm, entitled MergeAlign, that proposed to optimise a score of this type, but as shown in Additional file 1: Figure S5, it is possible to construct counter-examples for which the algorithm does not compute the optimal solution. As we shall illustrate, this lack of optimality can result in significant losses when summarising a set of alignments. Moreover, the same objective, i.e. penalising longer alignments, can be achieved through the use of a non-zero *g* parameter as described above, such that the use of modeller variant loss functions is unnecessary.

## Efficient data structures

In representing the alignment DAG, it is essential to ensure that the space complexity of the data structure is less than the total number of paths through the graph, which increases very rapidly with the number of columns. The obvious way to represent a graph is via a list of neighbours for each node, which requires $\mathcal {O}(\bar {d} |\Xi |)$ storage, where |*Ξ*| is the number of observed columns and $\bar {d}$ is the average node in-degree.

However, within the mean-field setting, we can use the predecessor and successor equivalence classes to significantly increase the space efficiency, since each column need only record its predecessor and successor equivalence class. Given the definitions of the predecessor and successor equivalence classes, we can see that each equivalence class is of size at most 2^*N*^−1, where *N* is the number of sequences, since each row can take one of two possible values (gap/character) in each equivalence class, with the restriction that the column cannot be all gaps. In general, the number of equivalence classes is therefore somewhat less than the number of columns, with $|\Xi | = \bar {d} |\mathcal {E}|$, where $1 \leq \bar {d} \leq 2^{N} - 1$. Using an equivalence-class representation of the DAG structure therefore results in $\mathcal {O}(\bar {d} |\mathcal {E}|) = \mathcal {O}(|\Xi |)$ space requirements, saving a factor of $\bar {d}$.

Similar gains can be made in time complexity. Since any column in a particular *f*_*P*_-equivalence class will have the same set of possible predecessors, and similarly for successors, the partial sums required in dynamic programming algorithms can be stored per equivalence class rather than per node, which results in algorithms of $\mathcal {O}(|\Xi |)$ time complexity rather than $\mathcal {O}(\bar {d} |\Xi |)$ (*see Algorithms 1 and 2 for examples*). In the limit of a large number of short sequences with high uncertainty, this results in going from approximately quadratic time, to time linear in the number of columns.

## Example application: summary alignments for simulated and benchmark datasets

In order to illustrate the utility of the aforementioned procedure, we first simulated sequence data using the program DAWG [117], yielding sets of sequences for which the true alignment is known. Details of the simulation are provided in Additional file 1: Section S7. Data were simulated under three parameter regimes, with indel rates set to low, medium and high (*see Additional file* 1 *: Section S7 for further details*); 50 datasets were generated for each regime, yielding 150 datasets overall, each containing 10 sequences, with average sequence length equal to 905 nucleotides.

As a biologically relevant example, we also considered a set of 78 alignments taken from the BAliBASE database, comprising the full-length alignments from the Reference 1 set [118]. This set further comprises two subsets, consisting of low sequence identity (Ref 1a, ID <25*%*) (short: 14, medium: 12, long: 12; average 6.8 sequences per alignment; average sequence length 309), and medium sequence identity (*Ref 1b*, ID =20−40*%*) (short: 14, medium: 16, long: 10; average 9.0 sequences per alignment; average sequence length 351). The simulated and BAliBASE datasets can be found in Additional file 2.

For each of these datasets, we ran the statistical alignment software StatAlign [56], which jointly samples alignments and trees under a stochastic model of substitution, insertion and deletion [93]. 1000 alignment samples were generated from the posterior distribution, and a Java-based implementation of Algorithm 2 was used to compute a summary alignment minimising the risk under the *C*- and *C* ^+^-based loss functions.

It is also of interest to consider how the minimum-risk summary approach scales to alignments containing larger numbers of sequences. As a test dataset containing larger alignments, we selected one of the largest alignments from the OXBench suite [119], consisting of 122 immunoglobulin sequences, with average length 113. To assess how the method scaled with the number of sequences after controlling for other factors (such as amino acid content and sequence length), we subsampled smaller datasets from this alignment, yielding datasets with 15, 33, 60 and 122 sequences. These subsets were sampled so as to maximise dissimilarity within the subset, since the original alignment contained several well-defined subgroups that would otherwise skew the analysis. Since full posterior sampling of alignments is only feasible for around 20-30 sequences, we made use of an approximate method for sampling alignments for these datasets [80], generating 2000 alignment samples for each dataset (*see Additional file* 1 *: Section S7 for further details*).

### Comparison to other methods

For comparison, we also generated summary alignments for each dataset using the MergeAlign method of Collingridge and Kelly [79], and a consistency-based approach whereby the alignment samples are used as a library for input to the program T-Coffee [114], using the -aln option [120]. We call the latter approach S-Coffee, with the ‘S’ signifying that the T-Coffee method is being used on a library derived from a set of sampled alignments.

As shown in Table 1, our DAG-based implementation is substantially faster than the other methods. Increasing the indel rate results in higher alignment uncertainty and longer alignments, resulting in an increase in runtime for all methods, although the increase is small for the minimum risk algorithm (henceforth referred to as MinRisk). Minimising the risk under the *C* ^+^-based loss function incurs an additional overhead due to the time needed to compute the weighted marginal probabilities, $\phantom {\dot {i}\!}p_{C^{+}}(X)$, but this takes less than half a second in all the examples we considered here.

**Table 1:** **Average time (in seconds) taken to generate a summary alignment from 1000 samples, for the three simulated datasets**

	**Indel rate**		
	***Low***	***Medium***	***High***
MinRisk (*C*)	1.5	1.8	2.2
MinRisk (*C* ^+^)	1.9	2.4	2.8
MergeAlign	12.0	17.6	22.9
S-Coffee	43.0	48.4	50.9

All tests performed on a single AMD Opteron 2.3GHz core.

### Accuracy metrics

To assess the performance of each approach, we make use of several measures of alignment accuracy, including the AMA metric of Schwartz [112,121] (measuring the proportion of correct pairwise homology statements), and the column score (equivalent to the *C* ^+^-score, measuring the proportion of correct columns). In addition, we use the measures shown in Table 2.

**Table 2:** **Accuracy measures used to assess the relative performance of the different summary methods**

**Name**	**Notation**	**Definition**
*C*-score	$\alpha _{C}(\hat {A})$	
Modeller *C*	${\alpha ^{m}_{C}}(\hat {A})$	
*C* ^+^-score	$\alpha _{C^{+}}(\hat {A})$	
Modeller *C* ^+^	$\alpha ^{m}_{C^{+}}(\hat {A})$	

*A* denotes the true alignment and $\hat {A}$ an estimated alignment, and |*A*| represents the length of alignment *A*.

For the simulated data, accuracy is computed relative to the known true alignments, and for the BAliBASE datasets, relative to the benchmark alignment provided.

Since the minimal $\mathcal {R}_{C}$ and $\mathcal {R}_{C^{+}}$ alignments maximise the expectation of the *C*- and *C* ^+^-score respectively, it would be expected that these methods perform best under the corresponding scores. The MergeAlign method seeks to maximise the Modeller *C* score, although as mentioned earlier, the algorithm cannot guarantee an optimal solution. As a pairwise progressive algorithm, the S-Coffee method might be expected to perform best under a sum-of-pairs score, such as the AMA metric.

Given that the absolute value of the accuracy varies substantially over the different datasets, we measure the performance of each method by computing a rank score, which indicates the rank of the accuracy of an alignment, $\hat {A}$, relative to the 1000 samples used as an input ()
(21)


A rank of 1 therefore indicates an alignment that is more accurate under measure *α* than each of the individual samples, whereas a rank of 0 indicates an accuracy lower than any of the individual samples.

### Results: simulated data

As shown in Table 3, the MinRisk method generally yields summary alignments that are more accurate than the majority of the samples, resulting in a rank score close to 1. As expected, minimising the risk under the *C*-based loss function results in the highest accuracy under metric *α*_*C*_, and similarly minimising the risk under $\phantom {\dot {i}\!}\mathcal {R}_{C^{+}}$ results in the highest scores under measure $\phantom {\dot {i}\!}\alpha _{C^{+}}$. Interestingly, the MinRisk *C* ^+^ method also results in the highest accuracy under the AMA sum-of-pairs metric. In all cases setting *g*=0 results in the best performance, since these accuracy metrics do not penalise false positives, although setting *g*=0.5 does not result in a large loss of performance.

**Table 3:** **Average rank scores for the different methods on simulated datasets, using the accuracy metrics described in the main text and in Table** 2

	***Low indel rate***			***Medium indel rate***			***High indel rate***		
	***α***_***C***_	$\protect \phantom {\dot {i}\!}\boldsymbol {\alpha _{C^{+}}}$	**AMA**	***α***_***C***_	$\protect \phantom {\dot {i}\!}\boldsymbol {\alpha _{C^{+}}}$	**AMA**	***α***_***C***_	$\protect \phantom {\dot {i}\!}\boldsymbol {\alpha _{C^{+}}}$	**AMA**
MinRisk (*C*), *g*=0	**0.91**	0.89	0.90	**0.96**	0.92	0.93	**0.89**	0.88	0.88
MinRisk (*C*), *g*=0.5	0.89	0.73	0.84	0.93	0.50	0.78	0.84	0.09	0.40
MinRisk (*C*), *g*=1	0.88	0.63	0.80	0.90	0.30	0.65	0.79	0.03	0.28
MinRisk (*C* ^+^), *g*=0	0.86	**0.98**	**0.96**	0.87	**1.00**	**1.00**	0.76	**1.00**	**1.00**
MinRisk (*C* ^+^), *g*=0.5	0.89	0.92	0.92	0.93	0.94	0.94	0.86	0.94	0.94
MinRisk (*C* ^+^), *g*=1	0.89	0.84	0.88	0.91	0.74	0.85	0.83	0.34	0.55
MergeAlign	0.65	0.40	0.48	0.80	0.46	0.58	0.73	0.36	0.45
S-Coffee	0.08	0.02	0.10	0.15	0.01	0.10	0.29	0.00	0.04

Highest values for each column shown in bold.

In contrast, on these datasets MergeAlign typically yields a summary alignment whose accuracy is close to the median, with a rank score close to 0.5, although performance is more reasonable under the *α*_*C*_ measure. The progressive heuristic S-Coffee algorithm performs consistently badly in all cases, yielding summary alignments that are typically worse than the majority of the samples used to build the library, suggesting a conflict between the information contained in the samples, and the heuristics used to construct the alignment.

**Figure 12:**
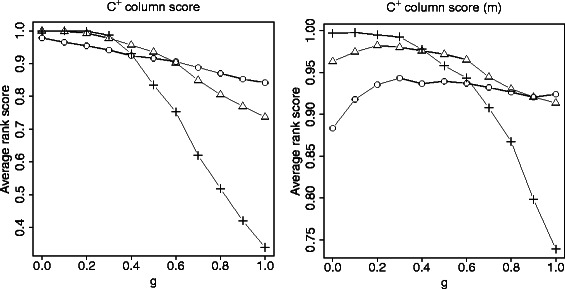
Accuracy as a function of the *g* parameter. Accuracy on the simulated datasets under the $\protect \phantom {\dot {i}\!}\alpha _{C^{+}}$ (left) and $\protect \phantom {\dot {i}\!}\alpha ^{m}_{C^{+}}$ (right) measures as a function of the *g* parameter for low (∘), medium (△) and high (+) indel rates.

When the modeller variants of the scores are considered (Table 4), the general patterns stay much the same, although there is now a benefit observed in increasing the *g* parameter, since the modeller scores penalise longer alignments. For alignments with more gaps (higher indel rate), the value of *g* yielding the highest accuracy under the modeller scores tends to decrease (*see Figure* 12). This reflects the fact that for cases where the true alignment contains many gaps we may wish to be more lenient with the inclusion of additional columns, allowing the alignment to increase in length. Overall, setting *g*=0.5 yields the best average performance under the modeller variants, corresponding to a loss function that equally penalises false positives and false negatives.

**Table 4:** **Average rank scores for the different methods on simulated datasets, measured using the modeller scores**

	***Low indel rate***		***Medium indel rate***		***High indel rate***	
	$\boldsymbol {{\alpha ^{m}_{C}}}$	$\boldsymbol {\alpha ^{m}_{C^{+}}}$	$\boldsymbol {{\alpha ^{m}_{C}}}$	$\boldsymbol {\alpha ^{m}_{C^{+}}}$	$\boldsymbol {{\alpha ^{m}_{C}}}$	$\boldsymbol {\alpha ^{m}_{C^{+}}}$
MinRisk (*C*), *g*=0	0.92	0.91	0.96	0.95	0.89	0.92
MinRisk (*C*), *g*=0.5	0.93	0.88	**0.97**	0.80	**0.90**	0.35
MinRisk (*C*), *g*=1	**0.95**	0.85	0.96	0.65	0.87	0.23
MinRisk (*C* ^+^), *g*=0	0.69	0.88	0.62	0.96	0.56	**1.00**
MinRisk (*C* ^+^), *g*=0.5	0.90	**0.94**	0.95	**0.97**	0.88	0.96
MinRisk (*C* ^+^), *g*=1	0.93	0.92	0.95	0.91	0.88	0.74
MergeAlign	0.74	0.57	0.85	0.67	0.78	0.63
S-Coffee	0.15	0.05	0.22	0.03	0.37	0.00

Highest values for each column shown in bold.

As might be expected, the performance of MergeAlign improves when the accuracy is measured using the modeller scores. However, better performance can still be obtained under the modeller variants by using the MinRisk method and a non-zero *g* parameter (*see Table* 4). As discussed earlier, the *g* parameter accomplishes the key aim of the modeller score (i.e. to penalise longer alignments) while maintaining computational tractability, and a meaningful statistical interpretation.

**Figure 13:**
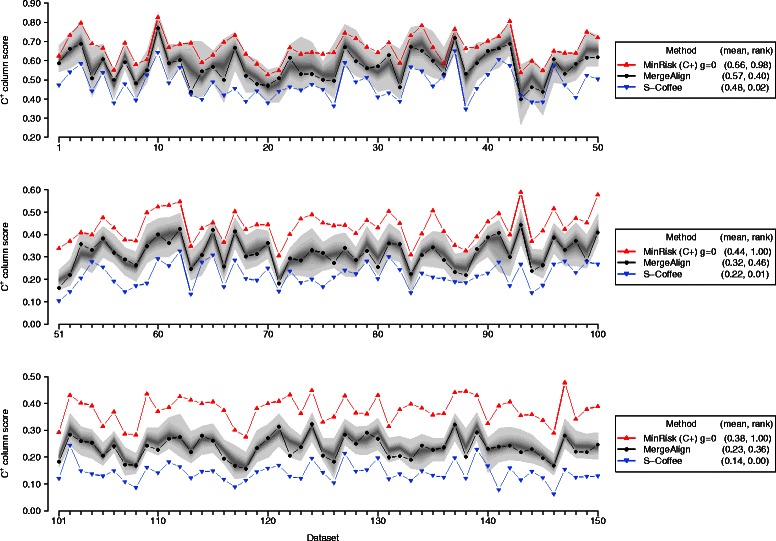
Accuracy of summary alignments for simulated data. Results for the MinRisk, MergeAlign and S-Coffee methods shown in red, black and blue respectively, for low (top panel), medium (middle panel) and high (bottom panel) indel rates, with accuracy measured by $\protect \phantom {\dot {i}\!}\alpha _{C^{+}}$. The range of values covered by the 1000 samples is shown in grey, with lighter shading indicating greater distance from the median.

Given the heterogeneity of the different datasets, it is also useful to visualise the results for the individual datasets. As shown in Figure 13 and Additional file 1: Figure S8, the results are consistent across all datasets, with the MinRisk method yielding alignments that are significantly better than the majority of samples, especially as the indel rate is increased. Conversely, the MergeAlign method consistently yields summary alignments that are close to the median accuracy of the sampled alignments, and the S-Coffee method performs consistently worse than the majority of samples.

### Results: BAliBASE

For the BAliBASE datasets, the MinRisk method also consistently yields summaries that are better than the majority of samples, and outperforms the other methods examined here in all cases (*see Tables* 5 *and* 6). Nevertheless, although still ranking behind most of the MinRisk combinations, MergeAlign performs somewhat better on the BAliBASE datasets than on the simulated data, with ranks scores consistently much higher than the median. This suggests that these particular BAliBASE alignments contain fewer of the types of features (for example large numbers of indels) that are likely to lead to suboptimal solutions under the MergeAlign algorithm. Similarly, the S-Coffee method, although still often worse than the median accuracy of the samples, performs better than on the simulated data, suggesting that the heuristics employed by T-Coffee are tailored more towards aligning these types of datasets. These heuristics may to some extent be overriding the information input via the library, which may explain the poor performance on the simulated datasets.

**Table 5:** **Average rank scores for the different methods on BAliBASE datasets, using the accuracy metrics described in the main text and in Table** 2

	***Ref 1a*** **(<25** ***%*** **)**			***Ref 1b*** **(20−40** ***%*** **)**		
	***α***_***C***_	$\phantom {\dot {i}\!}\alpha _{C^{+}}$	**AMA**	***α***_***C***_	$\phantom {\dot {i}\!}\alpha _{C^{+}}$	**AMA**
MinRisk (*C*), *g*=0	**0.94**	0.77	**0.88**	0.88	0.85	0.82
MinRisk (*C*), *g*=0.5	0.90	0.41	0.66	0.92	0.81	0.90
MinRisk (*C*), *g*=1	0.88	0.41	0.63	**0.94**	0.83	**0.93**
MinRisk (*C* ^+^), *g*=0	0.67	**0.92**	0.77	0.71	0.87	0.66
MinRisk (*C* ^+^), *g*=0.5	0.86	0.86	**0.88**	0.85	**0.91**	0.89
MinRisk (*C* ^+^), *g*=1	0.88	0.64	0.78	0.90	0.88	**0.93**
MergeAlign	0.91	0.59	0.74	0.80	0.75	0.84
S-Coffee	0.45	0.14	0.26	0.52	0.32	0.52

Highest values for each column shown in bold.

**Table 6:** **Average rank scores for the different methods on BAliBASE datasets, measured using the modeller scores**

	***Ref 1a*** ** (<25** ***%*** **)**		***Ref 1b*** ** (20−40** ***%*** **)**	
	$\alpha ^{m}_{C}$	$\alpha ^{m}_{C^{+}}$	$\alpha ^{m}_{C}$	$\alpha ^{m}_{C^{+}}$
MinRisk (*C*), *g*=0	0.93	0.74	0.82	0.78
MinRisk (*C*), *g*=0.5	**0.95**	0.70	0.96	0.96
MinRisk (*C*), *g*=1	0.92	0.68	**0.97**	**0.97**
MinRisk (*C* ^+^), *g*=0	0.40	0.50	0.34	0.33
MinRisk (*C* ^+^), *g*=0.5	0.86	**0.88**	0.83	0.85
MinRisk (*C* ^+^), *g*=1	0.90	0.86	0.93	0.96
MergeAlign	0.93	0.74	0.85	0.86
S-Coffee	0.59	0.46	0.76	0.75

Highest values for each column shown in bold.

We can see also that in general the optimal value of *g* for the MinRisk method is higher for the Ref 1b dataset reflecting the fact that these sequences are less diverged, and hence likely to contain fewer indels. However, as with the simulated data, a value of *g*=0.5 gives results that are close to optimal in all scenarios with the BAliBASE datasets.

### Results: approximate sampling on larger OXBench alignments

Using the OXBench datasets, we can examine how the above conclusions scale to alignments with larger numbers of sequences. As discussed by Bucka-Lassen *et al.* [95], the number of intersections between sampled alignments may be expected to decrease as the number of sequences is increased, due to the increased size of the state space. Similarly, since the number of possible columns increases exponentially with the number of sequences, it might be expected that the marginal probabilities of each column would decrease as the number of sequences is increased, thereby making the minimum-risk alignment less reliable.

However, in the examples considered here, this effect does not appear to be significant, since the alignment uncertainty also decreases as more sequences are added to the alignment, and this appears to more than compensate for the increase in the size of the potential state space (*see Table* 7). This is also highlighted by the fact that the average number of columns per equivalence class—a measure of the uncertainty surrounding the minimum-risk alignment—does not increase as the number of sequences is increased.

**Table 7:** **Results on OXBench datasets**

**Number of sequences**	**15**	**33**	**60**	**122**
Benchmark alignment length	144	150	152	157
Mean eq. class size	15.2	11.8	12.4	11.1
Average marginal	0.19	0.21	0.25	0.23
MinRisk rank, *g*=0	0.67	0.85	0.84	0.92
MinRisk rank, *g*=0.5	0.85	0.95	0.69	0.74
# columns in DAG	20288	26782	26221	30305
Time to read alignments (s)	0.5	0.8	1.2	2.1
Total runtime (s)	0.9	1.3	1.9	3.0

Timings were carried out using a single AMD Opteron 2.3GHz core.

**Figure 14:**
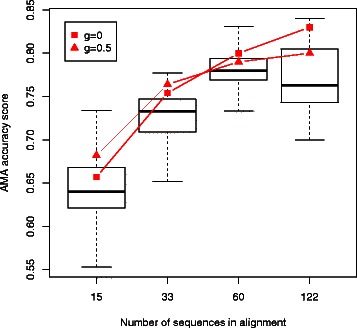
Accuracy as a function of the *g* parameter. Distribution of alignment accuracy scores for the OXBench datasets. Minimum-risk summary alignments shown in red, for *g*=0 and *g*=0.5. The summary alignments are generally more accurate than the majority of samples, and this remains the case as the number of sequences is increased.

As shown in Figure 14, although the marginal probabilities derived by the approximate sampling procedure may be less accurate than those from alignments obtained using StatAlign, the minimum-risk alignment for these alignments is still always better than the majority of samples, with a rank score often above 0.8 (*see Table* 7).

Since the alignments are of length around 150, and the DAGs contain in the region of 30,000 unique columns, 2000 samples is approximately 10 observations per column. While this appears to be sufficient for estimating the minimum-risk alignment, more samples will be needed in order to accurately estimate the probabilities of the less likely alignments, since these tend to converge more slowly (*cf. Figures* 7 *and* 8).

Overall the rank scores are of comparable magnitude to those observed with the BAliBASE datasets. Moreover, the performance does not appear to degrade as the number of sequences is increased, although the optimal value of *g* does switch from 0.5 to 0 as the number of sequences is increased to 60 and 122. This is likely due to the fact that the benchmark alignment increases in length as the number of sequences is increased, and a lower value of *g* favours longer alignments.

### Computational considerations

While the runtime does increase with the number of sequences, a breakdown of the contributions to these timings shows that the majority of the time is spent reading in the alignments, which scales linearly with the number of alignments multiplied by the number of sequences (*cf. Additional file* 1 *: Figure S9*). As discussed earlier, the minimum-risk algorithm scales linearly with the number of columns in the DAG, but this step contributes a very small proportion of the total runtime in the examples shown in Table 7. On our test systems the overall time taken to process and summarise 2000 alignments is only 3 seconds for the 122-sequence dataset (*see Table* 7), and around 10 seconds for 10,000 alignments (data not shown). For a 20-sequence dataset, analysing 500,000 alignments takes 150 seconds (*see Additional file* 1 *: Figure S9*). Memory usage is also generally low, requiring less than 2Gb in all the cases we have tested, even for 500,000 alignments.

In all cases we have examined, the time taken to actually generate the alignment samples is significantly larger than the time required to analyse the samples. As such, large gains in efficiency can be obtained by generating one set of alignment samples and carrying out multiple downstream analyses on this same set, compared to carrying out a full joint sampling analysis.

### Effect of alignment accuracy on tree estimation

As discussed in the introduction, a number of studies have highlighted how biases in alignments may lead to misleading conclusions in the context of downstream tree inference. As such, any methodology that has the potential to improve alignment accuracy, particularly in the presence of high uncertainty, has the potential to improve subsequent phylogenetic inference. Here we will provide a brief example to reiterate this point.

For each of the simulated datasets discussed earlier, we performed tree inference using the program DNAML from version 3.69 of the PHYLIP package [122], using alignments generated by four commonly used programs, as well as the summary alignments generated using the minimum-risk procedure presented here. DNAML was run with the default settings in each case, and the distance to the known true tree was computed using the Robinson-Foulds distance, equal to the number of bipartitions that differ from the true tree, with maximum value of 2(*n*−3), where *n* is the number of leaves in the tree [123].

**Figure 15:**
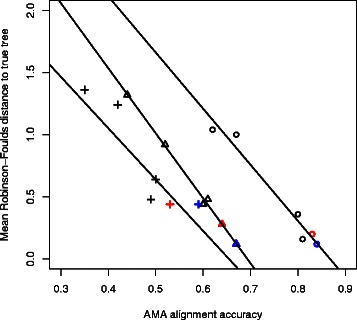
Alignment accuracy is strongly correlated with the number of errors in trees estimated by DNAML. Tree accuracy was measured using the Robinson-Foulds distance [123]. Results are shown for low (∘), medium (△) and high (+) indel rates, for the different methods presented in Table 8. In each case, the MinRisk results are highlighted in red (MinRisk *C*), and blue (MinRisk *C* ^+^), and tend to give the most accurate alignments and trees.

As shown in Table 8 and Figure 15, the alignment accuracy under these different methods correlates strongly with the accuracy of the resulting trees, with the most accurate alignment methods giving rise to the fewest tree errors. In all cases, the *C* ^+^ version of the minimum-risk algorithm, applied to alignments generated by StatAlign, yields the highest tree accuracy. This example illustrates the types improvements that can be obtained by using more robust methods to generate alignments before carrying out tree inference.

**Table 8:** **Results for tree inference on alignments generated using different methods, on the simulated datasets, as shown in Figure** 15

	***Low***		***Medium***		***High***		
	**AMA**	**RF**	**AMA**	**RF**	**AMA**	**RF**	
MinRisk (*C* ^+^), *g*=0	**0.84**	**0.12**	**0.67**	**0.12**	**0.59**	**0.44**	
MinRisk (*C*), *g*=0	0.83	0.20	0.64	0.28	0.53	**0.44**	
MAFFT	0.81	0.16	0.60	0.44	0.50	0.64	
MUSCLE	0.80	0.36	0.61	0.48	0.49	0.48	
T-Coffee	0.67	1.00	0.52	0.92	0.42	1.24	
CLUSTALW2	0.62	1.04	0.44	1.32	0.35	1.36	

Shown are alignment accuracy scores (according to the AMA metric), and Robinson-Foulds tree distances (RF) for the DNAML tree, averaged over all datasets in each group (low, medium and high indel rates). Higher alignment accuracy is strongly predictive of tree accuracy, with the most accurate alignments generating the trees with the fewest errors (shown in bold). The MinRisk results were computed using samples generated by StatAlign.

### Predictive power of column marginals

As well as providing a way to approximate full alignment probabilities, posterior column marginal probabilities can also be good predictors of the presence or absence of a column in the true alignment [22]. In all cases examined here, the column marginals are excellent predictors of the presence or absence of the column in the true alignment, with an AUC close to 1, especially for the BAliBASE datasets (*see Table* 9). The *C* ^+^-weighted marginals (the marginal probability of a column after grouping with all other columns containing the same characters, regardless of position in the alignment) are less accurate in predicting the presence/absence of a column under the *C* ^+^ definition, which may be due to the fact that the estimates of $\phantom {\dot {i}\!}p_{C^{+}}$ make stronger assumptions about the exchangeability of columns, averaging over a larger set of possible predecessors. In all cases, predictive power is higher for alignments containing fewer indels, although the predictive power of the marginals will depend largely on the suitability of the evolutionary model for analysing the dataset.

**Table 9:** **Accuracy of marginal probabilities in predicting column presence/absence, as measured by the area under a ROC curve (AUC), including a comparison to results generated using the program GUIDANCE [** 76 **] (indicated by the** ***p***_***G***_ **row in the table)**

	**Simulated data**			**BAliBASE**	
	***Low***	***Medium***	***High***	***Ref 1a***	***Ref 1b***
	0.93	0.92	0.90	0.99	0.99
	0.80	0.78	0.82	0.92	0.93
	0.84	0.78	0.75	0.79	0.89

Comparison to results generated by the widely-used program GUIDANCE [76] indicate that column marginals are typically a more reliable predictor of column presence/absence. However, it is important to note that the predictive power of these column marginals is dependent on the quality of the alignments used to construct the DAG.

## Propagating alignment uncertainty into downstream inference

So far we have examined how the DAG facilitates the efficient generation of accurate summary alignments, which can then be used for subsequent analyses. However, for many types of analyses it may be advantageous to jointly sample alignments and other parameters of interest, such as trees [56,57], or sequence annotations [65], in order to account for the interdependence of these different quantities. Since joint sampling approaches are typically computationally intensive, it is also desirable to explore alternative ways in which alignment uncertainty can be incorporated into downstream inference in cases where joint analysis is not feasible [29,124].

### Sequential approach

One way of accomplishing this is to carry out the downstream analyses separately on each of the sampled alignments, averaging or summarising the results as appropriate. This type of *sequential* approach has been used to assess the sensitivity of phylogenetic inference to the starting alignment [26,29,33], as well as examining the effect of alignment uncertainty on estimates of positive selection [36] and RNA secondary structure prediction [125].

However, as discussed earlier, a set of alignment samples will typically contain only a small portion of the total probability mass, even for pairwise alignments with relatively low uncertainty (*cf. Additional file* 1 *: Figure S3*). Hence, the uncertainty quantified in the individual samples will be a significant underestimate of the true alignment uncertainty.

Moreover, since the relative frequencies of whole alignments are a very poor estimator of posterior probabilities, simply carrying out an independent analysis on each sampled alignment and then averaging is likely to yield unreliable results. Reweighting procedures such as those discussed by Blackburne and Whelan [36] are only feasible when the posterior probability of each alignment can be computed exactly, which is not the case for many models of interest.

### DAG-based approach

In order to address these issues, we can make use of the alignment DAG, making use of intersections between alignments to increase the effective sample size.

Due to the acyclic structure of the graph, it is possible to adapt many standard algorithms, such as forward-backward algorithms for HMMs, to operate on the DAG structure rather than an individual alignment. This allows for downstream inference to be averaged over a very large number of alignments, weighted according to a more reliable estimate of the posterior probability for each alignment, rather than analysing only a small collection of individual samples.

As a specific example, we can consider the case of tree inference under an independent-sites model. On a single alignment the posterior probability of a tree, *Υ*, can be written as a product of contributions from each column:

(22) $$\begin{array}{@{}rcl@{}} &p(\Upsilon \mid A, \Theta) \propto p(\Upsilon) \prod\limits_{i=1}^{L_{A}} p\left(A^{(i)} \mid \Upsilon, \Theta\right)  \end{array} $$

where *Θ* represents the parameters of the evolutionary model, and the proportionality involves the quantity $\int p(A, \Upsilon) d\Upsilon $. It is a straightforward extension then to compute the posterior averaged over all alignments in the DAG, using a dynamic programming approach similar to the algorithms discussed earlier. We first introduce the following partial sum for a column *X*:

(23) $$ z(X \mid \Upsilon, \Theta) \propto p(X \mid \Upsilon,\Theta) \sum\limits_{X^{\prime} \ltimes X} z(X^{\prime} \mid \Upsilon,\Theta) p(X \mid X^{\prime})  $$

such that the marginal posterior for the tree, *Υ*, summing over all alignments in a DAG $\mathcal {D}(\mathcal {A})$, can be written as

(24) $$\begin{array}{@{}rcl@{}} p(\Upsilon \mid \mathcal{D}(\mathcal{A}), \Theta) &\propto& p(\Upsilon) \sum\limits_{A \in \mathcal{D}(\mathcal{A})} p(A) p(\Upsilon \mid A, \Theta) \end{array} $$

(25) $$\begin{array}{@{}rcl@{}} &\propto& p(\Upsilon)\, z\big(X^{(T)}_{\mathcal{A}} \mid \Upsilon, \Theta\big) \end{array} $$

### Example application: marginal probabilities for topologies

As an illustration of the utility of this approach, we consider here a 4-sequence example, for which there are three possible unrooted topologies relating the sequences. The specific example we consider consists of three human globin sequences, *α*-haemoglobin (HbA), myoglobin (Mb), and cytoglobin (Cygb), as well as a plant leghaemoglobin (LegHb) (*datasets can be found in Additional file* 2). Previous studies have shown significant uncertainty as to the phylogenetic relationship between these different types of globins [62], hence this represents a good test case to analyse the effect of alignment uncertainty on topology inference. Here we restrict our analysis to four sequences for the purposes of simplifying the example.

For these sequences, a set of alignment samples, , and tree samples, , was generated using StatAlign (*see Additional file* 1 *: Section S7 for further details*), and the marginal likelihood for each tree in the set was then computed as a sum over all the alignments by evaluating the quantity $z(X^{(T)}_{\mathcal {A}} \mid \Upsilon, \Theta)$ for all $\Upsilon \in \mathcal {T}$. The parameters, *Θ*, were set using the Dayhoff substitution matrix [103], with gaps treated as missing data. Assuming a uniform prior, the marginal posterior probability for each topology, *τ*, was then computed by averaging the marginal likelihoods for all trees in  conforming to the particular topology:
(26)


where  indicates that tree *Υ* conforms to topology *τ*. These marginal posteriors can then be compared to the topology posterior computed on each alignment individually, replacing $\mathcal {D}(\mathcal {A})$ with *A* in Equation (26) above.

**Figure 16:**
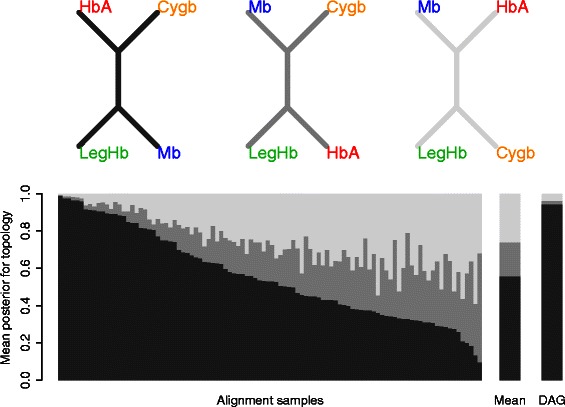
Posterior probabilities for three possible topologies, computed on individual alignment samples (bottom left), as well as marginalising over the alignments within the DAG (bottom right).*Top panel:* The three unrooted topologies for the four globin sequences discussed in the main text, ordered according to the posterior probability according to StatAlign (*left to right, descending in probability*). The leghaemoglobin sequence is taken from L.luteus, and all others from H.sapiens. *Bottom panel:* Posterior probabilities computed on individual alignment samples (*left*), and by marginalising over all alignments contained within the DAG (*right*). Bars in the lower panel are colour-coded according to the shading of the tree topologies in the top panel, and ordered according to the probability of the first topology. Also shown is the mean of the probability vectors computed on the individual alignment samples (*right*).

Although the true tree is not known in this case, the trees sampled by StatAlign place the majority of the posterior mass on the left-most topology shown in the top panel of Figure 16, placing a posterior probability of 0.12 on the centre tree, and 0.09 for the right-most topology.

The bottom panel of Figure 16 shows posterior probabilities computed using Equation (26), indicating significant variability depending on which alignment is used. While some alignments result in a posterior probability of more than 0.9 for the most favourable topology, others result in a probability of less than 0.2 for this topology. Simply taking the mean posterior over all the individual alignments in this case results in a posterior probability of only 0.56 for the most favourable topology. However, combining all the alignment samples into the DAG leads to a posterior probability of 0.94. This illustrates the fact that combining the alignments into a DAG may result in additional information being extracted from the same set of alignments, due to the increased effective sample size arising from intersections in the DAG.

Since the same DAG is used to compute the likelihood for all trees in the set , the majority of the runtime for this procedure is not spent reading in the alignments from disk (as it was for the minimum-risk summary procedure). As such, the runtime scales linearly with the number of columns in the DAG, as expected (*see Additional file* 1 *: Figure S10*).

## Conclusions

The approaches illustrated here provide a general framework for dealing with alignment uncertainty in a statistically meaningful fashion. Encoding a set of sampled alignments in a DAG structure allows for more accurate estimation of posterior probabilities based on column or pair marginals. Due to interchanges and crossovers in the DAG, the number of alignments encoded in the graph is typically many orders of magnitude greater than the number of samples used to generate the DAG, such that the effective sample size is greatly increased by this representation.

Since the graph is acyclic, efficient algorithms can be developed for summation over this very large number of alignments, each weighted according to its probability. As a specific example, we have considered algorithms for generating summary alignments that minimise the expected value of various types of loss functions, observing that this type of algorithm is generally very successful at minimising the loss on a set of test cases.

This approach provides a way to conduct many types of sequence analysis on the very large set of alignments encoded in the DAG structure, allowing for alignment uncertainty to be propagated into downstream inference in cases where computationally expensive joint sampling procedures are infeasible. In addition to the tree inference example illustrated here, we are currently working on adapting several other common algorithms to the alignment DAG structure.

### Combining the output of other alignment programs

The approaches detailed here are in theory applicable to a set of alignments generated by any type of method, although the quality of the probability estimates generated by the DAG will depend on the quality of the underlying model used to generate the alignments. Although this type of method can be used to combine the output of several different alignment programs, in a similar fashion to the M-Coffee procedure [120], such an approach does not have a probabilistic interpretation, and will depend heavily on the choice of programs used to generate the input.

We have observed that this type of procedure usually yields summary alignments that are similar in accuracy to the program that typically generates the most accurate alignments (data not shown); however, since the most accurate alignment method is usually known from the outset, based on benchmarking results, there is not much to be gained by employing such a procedure. Moreover, the reliability of such an approach as a heuristic will depend strongly on the degree of similarity between the different alignment programs, hence we would recommend against using alignment DAGs as a way of combining the output of non-probabilistic alignment programs.

### Alignment DAGs as generators of alignment samples

One other obvious application of the alignment DAG is as a way of generating additional alignment samples, which can be sampled by using a DAG-based version of the traditional stochastic traceback algorithm (*cf. Additional file* 1 *: Section S6*).

One potential use for these alignment samples could be as a source of proposals within an MCMC alignment sampler, allowing for a new state to be efficiently generated, along with a known proposal probability for use in a Metropolis-Hastings accept/reject step. Although this type of approach does not allow for the exploration of previously unobserved columns, it could be useful as way to improve mixing, particularly once the key regions of the space have already been explored.

## Software availability

Java software implementing the minimum-risk alignment summary algorithm and computation of marginal topology probabilities is available for download at http://statalign.github.io/WeaveAlign. A platform-independent jar archive containing version 1.2.1 of WeaveAlign is included in Additional file 2, along with datasets and example results.

## Additional files

Additional file 1
**Supplementary methods and figures.**

Additional file 2
**Software and datasets.**
