# Supplementary material for: Efficient representation of uncertainty in multiple sequence alignments using directed acyclic graphs
Source: BMC Bioinformatics. 2015 Apr 1;16:108. doi: 10.1186/s12859-015-0516-1 (PMC4395974; doi:10.1186/s12859-015-0516-1)
Supplement: Additional file 1 — Supplementary methods and figures. [file 12859_2015_516_MOESM1_ESM.pdf]

Efficient representation of uncertainty in multiple sequence alignments using directed acyclic graphs

Supplementary material

JL Herman *et al.* (2015)

S1 Estimation of conditional marginal probabilities from local alignments

As discussed in the main text, the conditional marginal probabilities for each column can also be estimated from a collection, $\mathcal{A}^+$, of local alignments sampled according to their probability. Unlike with the global alignment case discussed in the main text, a common normalising factor of $|\mathcal{A}^+|$ can no longer be used for all columns, such that the marginal probability for a column, $p(X)$, cannot be estimated from the collection of local alignments. However, due to the mutual exclusivity of the columns following a particular equivalence class as defined by equation (2) in the main text, the normalised conditional marginals can still be recovered from the expression

$$\hat{p}_C(X \mid \mathcal{P}(X)) = \hat{p}_C(X \mid E_P(X)) = \frac{n_C(X, \mathcal{A}^+)}{n_C(E_P(X), \mathcal{A}^+)} \tag{S1}$$

where $n_C(E_P(X), \mathcal{A}^+) = \sum_{X' \in E_P(X)} n_C(X', \mathcal{A}^+)$, and

$$n_C(X, \mathcal{A}^+) = \sum_{A \in \mathcal{A}^+} \mathbb{1}(C(X) \in C(A)) \tag{S2}$$

In the case where alignments are sampled by iteratively modifying subalignments, the efficiency of the estimators for the conditional marginals may be improved by considering the changed portion of the alignment as a local alignment sample, rather than counting the modified alignment as a new global alignment. We postpone a more thorough investigation of the properties of these different estimators for future research.

S2 Derivation of posterior risk

Beginning with the following general loss function as in equation (17) in the main text

$$\begin{aligned}\mathcal{L}_f(X \,\|\, A) =& \lambda_{FP}(1 - \mathbb{1}(f(X) \in f(A))) \\ & - \rho_{TP}\mathbb{1}(f(X) \in f(A))\end{aligned} \tag{S3}$$

$$= \lambda_{FP} - (\rho_{TP} + \lambda_{FP})\mathbb{1}(f(X) \in f(A)) \tag{S4}$$

the posterior risk can be written as

$$\begin{aligned}\mathcal{R}_f(A) &= \sum_{A'} p(A') \sum_{X\in A'} \lambda_{FP} - (\rho_{TP} + \lambda_{FP})\mathbb{1}(f(X) \in f(A)) \\ &= \sum_{A'} p(A') \sum_{X\in A} \lambda_{FP} - (\rho_{TP} + \lambda_{FP})\mathbb{1}(f(X) \in f(A')) \\ &= \sum_{j=1}^{L_A} \sum_{A'} p(A')[\lambda_{FP} - (\rho_{TP} + \lambda_{FP})\mathbb{1}(f(X) \in f(A'))]\end{aligned}$$

where the second line interchanges A and A', which relies on the bijective nature of f. Defining a weighted marginal probability under a function, f, as

$$p_f(X) = \sum_A p(A)\ \mathbb{1}(f(X) \in f(A)) \tag{S5}$$

this can be rewritten as

$$\mathcal{R}_f(A) = \sum_{j=1}^{L_A} \lambda_{FP} - p_f(A^{(j)})(\rho_{TP} + \lambda_{FP}) \tag{S6}$$

$$\propto \sum_{j=1}^{L_A} \frac{\lambda_{FP}}{(\rho_{TP} + \lambda_{FP})} - p_f(A^{(j)}) \tag{S7}$$

as presented in equation (18) in the main text.

S3 Pairwise loss functions

As mentioned in the main text, it is possible to describe several different types of pairwise accuracy scores using a loss function of the form

$$\mathcal{L}_{pw}(X \parallel A) = \sum_{i=1}^{N-1} \sum_{j=i+1}^{N} \rho_{TP}(X_i, X_j)\mathbb{1}((X_i, X_j) \in A) \tag{S8}$$

where N is the number of sequences. With

$$\rho_{TP}(X_i, X_j) = -\mathbb{1}(X_i \neq \text{gap})\mathbb{1}(X_j \neq \text{gap})$$

this is equivalent to the commonly used *sum-of-pairs* score [1], and the AMA alignment metric of Schwartz [2,3] can be obtained by setting

$$\begin{aligned}\rho_{TP}(X_i, X_j) = &- \mathbb{1}(X_i \neq \text{gap})\mathbb{1}(X_j \neq \text{gap}) \\ &- G_f\,\mathbb{1}(X_i = \text{gap})\mathbb{1}(X_j \neq \text{gap}) \\ &- G_f\,\mathbb{1}(X_i \neq \text{gap})\mathbb{1}(X_j = \text{gap})\end{aligned} \tag{S9}$$

where $G_f > 0$.

S4 Kullback-Liebler divergence of factored approximations

The deviation between the true distribution over alignments, $p(A)$, and an approximation, $q(A)$, can be measured using the Kullback-Liebler (KL) divergence

$$d(p \parallel q) = \sum_A p(A) \frac{\log p(A)}{\log q(A)} \tag{S10}$$

$$= \text{const.} - \sum_A p(A) \log q(A) \tag{S11}$$

Minimising the KL divergence for a fixed $p(A)$ is equivalent to maximising the *relative entropy*, $\sum_A p(A) \log q(A)$, subject to restrictions on the form for q.

For DAG-based representations, $q(A)$ can be factored along the edges of the DAG; writing the log of the product of conditionals as a sum of logs, the relative entropy can be written in the form

$$\sum_A p(A) \log q(A) = \sum_A p(A) \sum_X \sum_{X' \ltimes X} \mathbb{1}(X \in A)\mathbb{1}(X' \in A) \log q(X \mid X') \quad (S12)$$

$$= \sum_X \sum_{X' \ltimes X} p(X \mid X') \log q(X \mid X') \quad (S13)$$

Using a Lagrange multiplier to enforce the normalisation of $q(X \mid X')$, the distribution maximising equation (S13) satisfies the following equation, for all X, X'

$$0 = \frac{\partial}{\partial q(X \mid X')} \left[p(X \mid X') \log q(X \mid X') + \lambda \left(1 - \sum_{X'' \in E_P(X)} q(X'' \mid X') \right) \right] \quad (S14)$$

$$= \frac{p(X \mid X')}{q(X \mid X')} - \lambda \quad (S15)$$

such that the divergence is minimised with $q(X \mid X') = p(X \mid X')$, which corresponds to setting the pairwise distributions in equation (6) in the main text to be equal to the true pair marginals. This is equivalent to the result stated in Theorem 11.1 of Cowell *et al.* [4].

For the mean-field approximation, replacing $q(X \mid X')$ by $q(X \mid \mathcal{P}(X))$ in equation (S15), and summing over X', it is clear that the KL divergence is also minimised by writing these conditionals in terms of the corresponding marginal distributions:

$$q(X \mid \mathcal{P}(X)) = p(X \mid \mathcal{P}(X)) \quad (S16)$$

where $p(X \mid \mathcal{P}(X))$ is the probability of column X given that one of its possible predecessors is in the alignment (*cf. equation (8) in the main text*).

While in the more general context marginalisation over the distribution p may be intractable, in this work consider the empirical distribution, $\hat{p}(A)$, rather than constructing a parametric form for $p(A)$. Hence consistent marginals can be computed by simply taking the empirical marginals, without recourse to iterative procedures.

S5 Dynamic programming algorithm for summing over paths in the DAG

In order to compute the sum of probabilities for all alignments contained within the DAG, we first define the partial sum for column X as

$$z(X) = \begin{cases} \sum_{X' \ltimes X} z(X') p(X \mid X') & (\textit{pair marginals}) \\ z(E_P(X'))\; p(X)/p(E_P(X)) & (\textit{mean field}) \end{cases} \quad (S17)$$

where $z(X^{(0)}) = 1$ and

$$z(E_P(X)) = \sum_{X' \in E_P(X)} z(X') \quad (S18)$$

The total sum for the whole DAG is then given by $z(X^{(T)}_{\mathcal{A}})$, with $X^{(T)}_{\mathcal{A}}$ denoting the terminal column in the DAG $\mathcal{D}(\mathcal{A})$, as defined in the main text. This quantity can be computed in time and space linearly proportional to the number of columns in the DAG, in contrast to the $O(L^N)$ time and space taken for filling the full N-dimensional dynamic programming table. Replacing $p(X)$ with $\mathbb{1}(p(X) > 0)$ results in an algorithm for computing the number of paths through the DAG.

S6 Stochastic traceback through the DAG

The traditional stochastic traceback algorithm described by Durbin *et al.* [5] allows for alignments to be sampled according to their posterior distribution. However, this type of approach can only be used in cases where the full dynamic programming matrix can be filled out, limiting the application to alignments with larger numbers of sequences.

An analogous algorithm can be constructed to stochastically sample paths through the DAG, allowing for alignments to be sampled according to the posterior distribution described by the DAG. This simply involves building up an alignment according to the following recursive formula

$$p(A^{(i)} = X \mid A^{(i-1)} = X') = p(X \mid X')z(X')/z(X) \tag{S19}$$

where $z(X)$ is defined as in equation (S17), and $p(X \mid X')$ can be derived using either the pair-marginal or mean-field expression, as desired.

S7 Assembly and analysis of datasets

S7.1 Simulated alignments & BAliBASE

The dataset of simulated alignments used for assessing the minimum risk summary algorithm was generated using the sequence evolution simulation tool DAWG [6]. A random phylogeny of 10 sequences was chosen and fixed (*see Figure S6*), and sequences were simulated under the GTR substitution model (rates of substitution for AC, AG, AT, CG, CT and GT were set at 1.5, 3.0, 0.9, 1.2, 2.5 and 1.0; equilibrium frequencies for A, C, G and T were set at 0.20, 0.30, 0.30, 0.20), with the G+I rate heterogeneity model ($\gamma = 0.9$, $\iota = 0.05$), and an indel process with lengths distributed according to a negative binomial $NB(3, 0.7)$ distribution. The indel rate was set to three different values [0.01 (low), 0.02 (medium) and 0.03 (high)], to generate datasets of varying alignment uncertainty. For each indel rate, 50 alignments were generated, yielding 150 datasets overall.

The BAliBASE alignments were taken from subsets RV11 and RV12 of version 3.0 of the database. Both the simulated and BAliBASE datasets can be found in Additional file 2.

StatAlign v1.1 was run using the default settings for nucleotides (for the simulated data), and amino acids (for the BAliBASE data), with a burnin of 500,000, and 2 million sampling steps, taking alignment samples every 2000 steps, thus producing 1000 alignment samples for each test case.

S7.2 OXBench alignments

For the larger datasets, reference alignments were obtained from version 1.3 of OXBench, downloaded from
`www.compbio.dundee.ac.uk/downloads/oxbench/oxbench_1_3.tar.gz`
One of the largest alignments was chosen, found in the directory `oxbench_1_3/data/align/fasta/12`. In order to assess the affect of the number of sequences while controlling for other factors, we opted to analyse subsets of this alignment. To avoid ending up with subsets containing highly similar proteins corresponding to clades within the original set, we used a greedy

algorithm to choose maximally dissimilar sets of particular sizes, producing subsets of sequences of size 15, 33 and 60, in addition to the full set of 122 sequences. These alignment samples, along with an example script for computing the minimum-risk summary and alignment accuracy across the samples, are available at `https://github.com/statalign/WeaveAlign`.

Sets of alignments were generated using an approximate iterative MCMC procedure [7], generating 2000 samples for each dataset. This algorithm iterates between sampling of substitution matrices, and computation of the optimal score-based alignment using a program such as MUSCLE [8], generating a set of alignments according to an simplified posterior distribution.

S7.3 Globin alignments for marginal topology computations

The alignments used for computing the marginal topology probabilities were generated by running StatAlign v3.2 on four globin sequences (human cytoglobin, myoglobin and α-haemoglobin, as well as lupin leghaemoglobin) for $200,000$ iterations using the Dayhoff rate matrix, taking samples every 100 iterations. These 2000 alignment samples were then further thinned down by a factor of 20 to yield 100 alignments.

The marginal probability of each tree topology was computed by averaging over 500 tree samples, as discussed in the main text. These marginal probabilities were computed on each of these alignment samples individually, as well as on the DAG formed from the 100 alignment samples.

The script `marginal_tree_posterior_analysis/example-analysis.sh` in Additional file 2 carries out the above analyses using the program WeaveAlign (available at `http://statalign.github.io/WeaveAlign`, and also contained in Additional file 2), requiring 1-2 minutes on a 2.3GHz core. It should be noted that with the current version of the code the analysis of individual alignments requires the set of tree samples to be read in multiple times, hence some simple optimisations to the code would likely increase the efficiency of this process.

S7.4 Comparison to other alignment programs for downstream topology inference

For comparisons with MUSCLE, T-Coffee and CLUSTALW2 the default settings were used, and MAFFT was run with the `--auto` setting.

References

1. Thompson JD, Higgins DG, Gibson TJ: **CLUSTAL W: improving the sensitivity of progressive multiple sequence alignment through sequence weighting, position-specific gap penalties and weight matrix choice**. *Nucleic Acids Research* 1994, **22**(22):4673–4680.
2. Schwartz AS, Myers EW, Pachter L: **Alignment metric accuracy**. *arXiv:q-bio/0510052* 2005.
3. Schwartz AS: **Posterior decoding methods for optimization and accuracy control of multiple alignments**. *PhD thesis*, University of California, Berkeley 2007.
4. Cowell R, Dawid P, Lauritzen S, Spiegelhalter D: *Probabilistic networks and expert systems.* Information Science and Statistics, Springer, New York 2007.
5. Durbin R, Eddy SR, Krogh A, Mitchison G: *Biological Sequence Analysis : Probabilistic Models of Proteins and Nucleic Acids.* Cambridge University Press, Cambridge 1998.
6. Cartwright RA: **DNA assembly with gaps (DAWG): Simulating sequence evolution**. *Bioinformatics* 2005, **21**(Suppl 3):31–38.
7. Herman JL, Szabó A, Miklós I, Hein J: **Approximate posterior sampling of multiple sequence alignments by iterative perturbation of substitution matrices**. *arXiv:1501.04986* 2015.
8. Edgar RC: **MUSCLE: A multiple sequence alignment method with reduced time and space complexity**. *BMC Bioinformatics* 2004, **5**:113.
9. Mizuguchi K, Deane CM, Blundell TL, Overington JP: **HOMSTRAD: a database of protein structure alignments for homologous families**. *Protein Science* 1998, **7**(11):2469–2471.

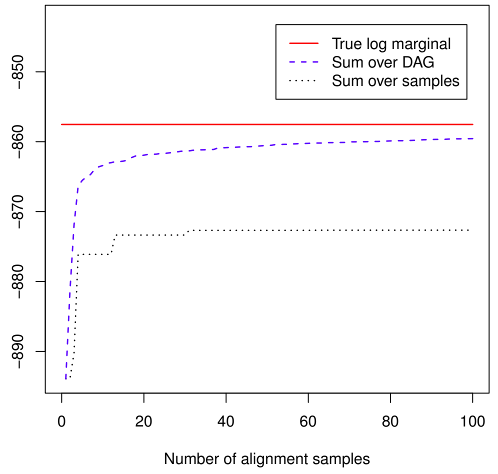

Figure S1 The probability mass contained within the individual samples increases relatively slowly, and encapsulates only a very small fraction of the total. In contrast, the proportion of the posterior mass encapsulated in the set of paths through the alignment DAG increases much more rapidly.

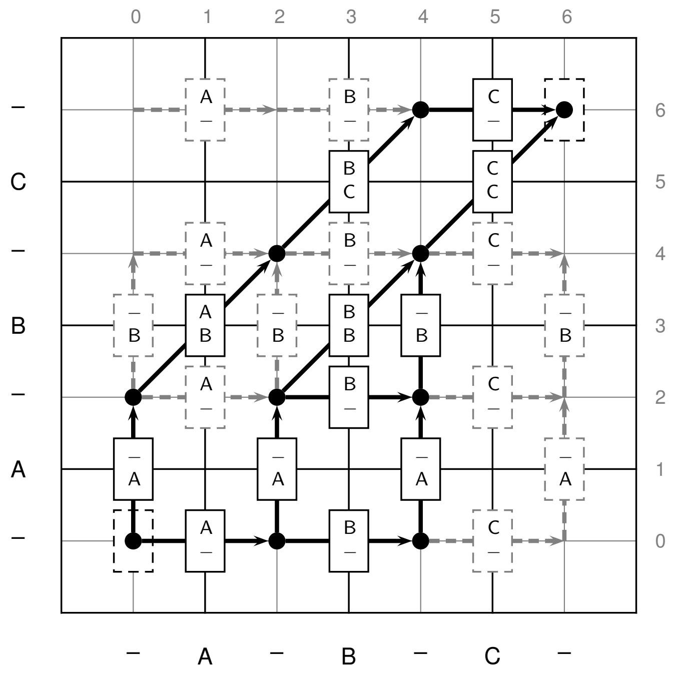

Figure S2 If gaps are not distinguished based upon their position in the alignment, it is effectively the same as replicating all gap-containing columns onto all parallels in the graph; in the pairwise case, this is equivalent to replicating each gapped column onto all horizontal and vertical parallels (shown by dotted grey columns and edges in the figure above). This means that the graph in general becomes maximally dense, such that the complexity of any algorithms scales in the same way as the full dynamic programming problem. In contrast, by differentiating between columns based upon where the gaps occur, a sparse graph is retained. NP-hardness of finding minimum-risk alignments among all valid column orderings under the C^{+} coding can be proven more rigorously by reduction from the Hamiltonian Cycle problem. We omit details here for brevity.

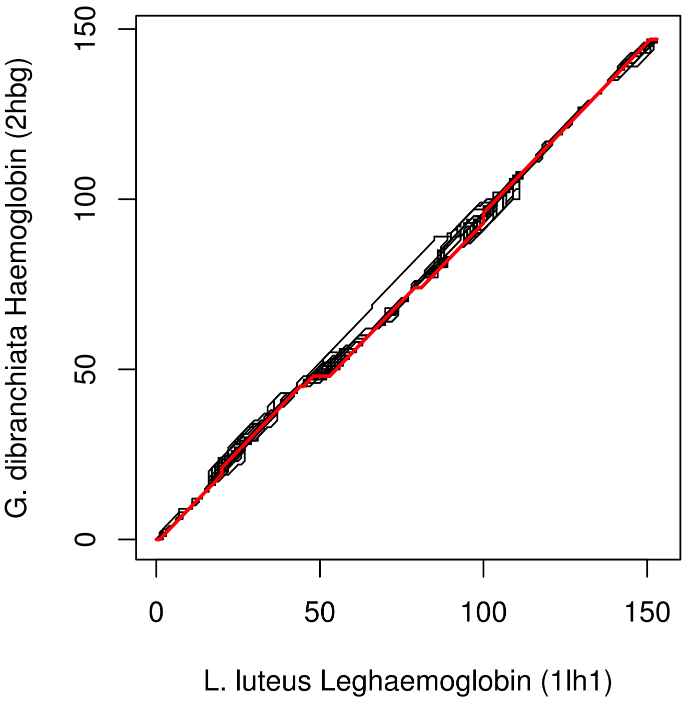

Figure S3 A set of 100 pairwise alignments sampled directly from the pair-HMM shown in Figure S4, for two globin sequences. Overlaid in red is the structural alignment taken from the HOMSTRAD database [9]. Despite strong similarity between the alignments, each sample is unique, such that it is not possible to estimate posterior alignment probability on the basis of whole alignment frequency.

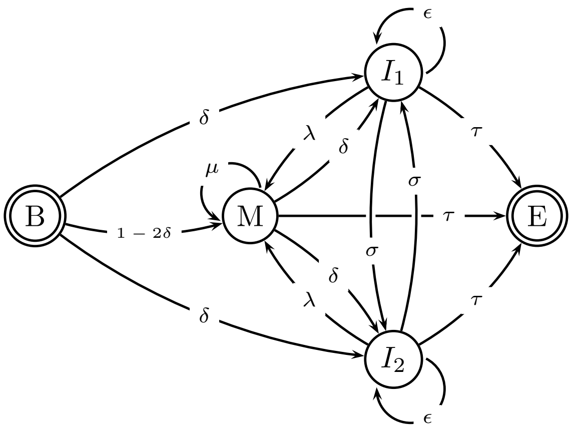

Figure S4 The pair-HMM used to sample pairwise alignments between the two globin sequences, as illustrated in Figure S3. The states correspond to: B = begin, E = end, M = match, I_1 = indel, and I_2 = indel. We have used the shorthand $\mu = 1 - 2\delta$ and $\lambda = 1 - \epsilon - \sigma - \tau$. For the analyses described in the text, we set $\delta = 0.03$ and $\epsilon = 0.3$, corresponding to an affine gap model; τ was set to the expected sequence length, i.e. $2/(L_1 + L_2)$. The parameter σ, representing the probability of independent adjacent insertions, was set to 0.1, reflecting the fact that insertions may be more common in certain regions of a protein, such as flexible loops. Very similar results were observed with small variations on these parameter values. Sampling was carried out using the algorithms described by Durbin *et al.* [5].

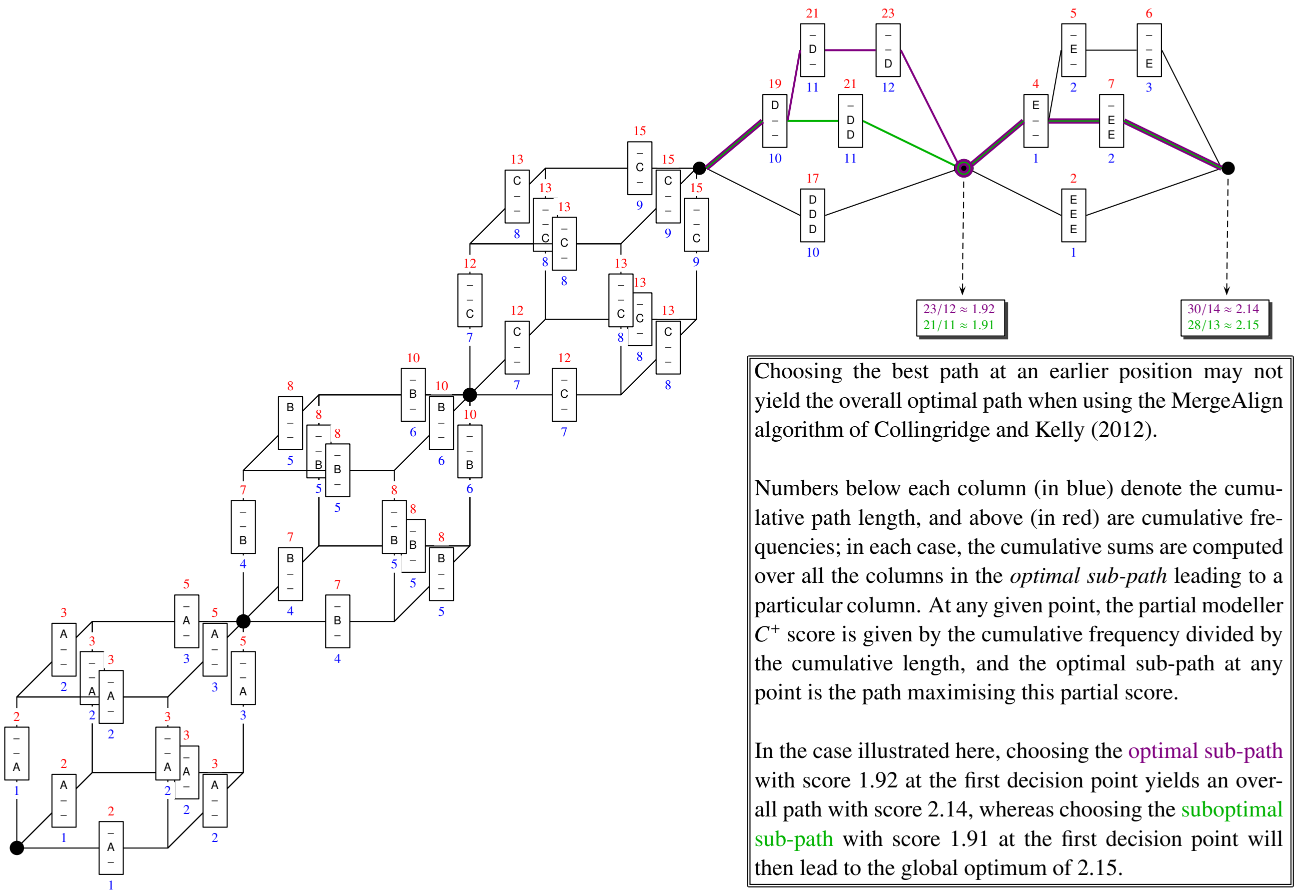

Figure S5 Example illustrating a case where the algorithm of Collingridge and Kelly does not yield the global optimum under the modeller (length-normalised) version of the *C*-score.

Choosing the best path at an earlier position may not yield the overall optimal path when using the MergeAlign algorithm of Collingridge and Kelly (2012).

Numbers below each column (in blue) denote the cumulative path length, and above (in red) are cumulative frequencies; in each case, the cumulative sums are computed over all the columns in the *optimal sub-path* leading to a particular column. At any given point, the partial modeller C^+ score is given by the cumulative frequency divided by the cumulative length, and the optimal sub-path at any point is the path maximising this partial score.

In the case illustrated here, choosing the optimal sub-path with score 1.92 at the first decision point yields an overall path with score 2.14, whereas choosing the suboptimal sub-path with score 1.91 at the first decision point will then lead to the global optimum of 2.15.

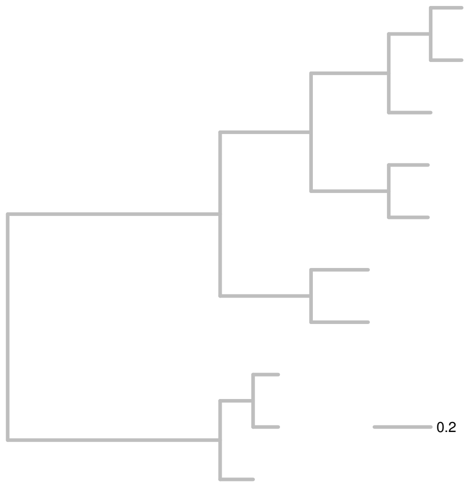

Figure S6 The tree used to generate simulated data as described in Section S7.

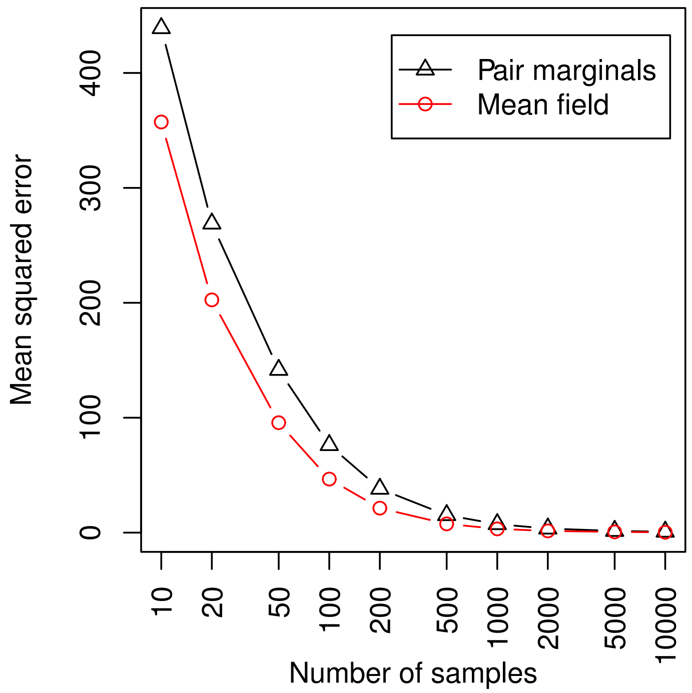

Figure S7 Mean squared error in the approximation to the true posterior, as a function of the number of alignment samples, for the pairwise globin example, with $\delta = \epsilon = \sigma$, such that the likelihood is completely site-independent. In this case, the mean-field, single-column marginal estimate always dominates the two-state, pair marginal estimate, due to the increased effective sample size.

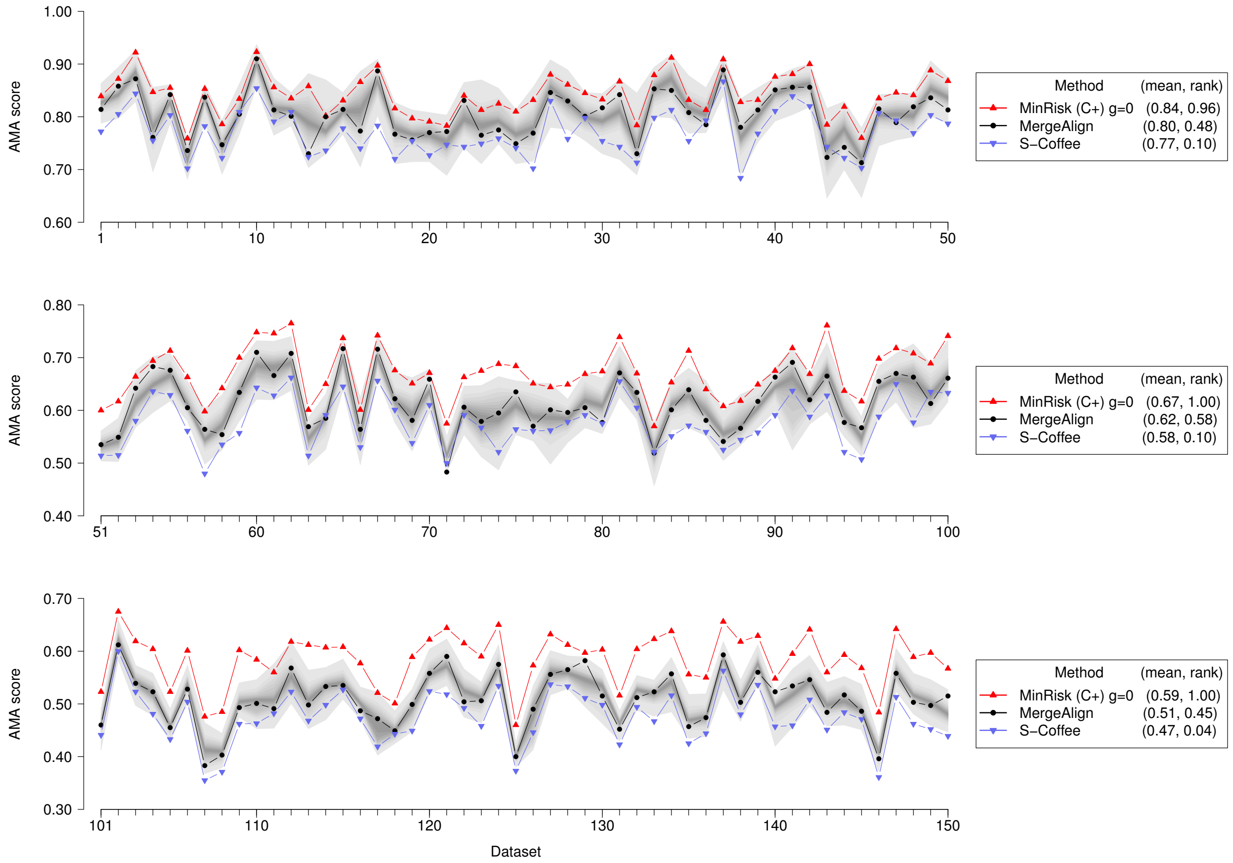

Figure S8 Accuracy of summary alignments for simulated data under three different methods as measured by α_{AMA}, for low (top panel), medium (middle panel) and high (bottom panel) indel rates. The range of accuracy values covered by the StatAlign samples is shown in grey, with lighter shading indicating greater distance from the median.

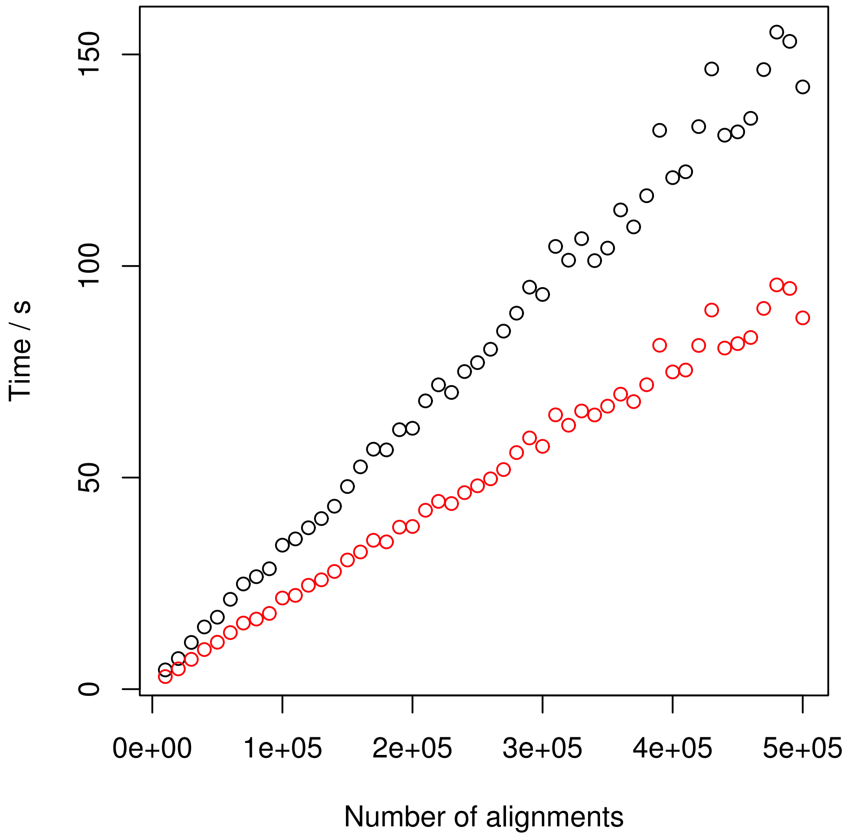

Figure S9 Total runtime (black) and time spent creating the DAG structure (red) when generating a minimum-risk summary alignment, as a function of the number of alignments used as input, showing the expected linear scaling. Results shown for alignments generated on 20 globin sequences, timed on a single AMD Opteron 2.3GHz core, on a system with 7200rpm disks. It should be noted that the figures here include the time for creation of the DAG and execution of the minimum-risk summary algorithm, but do not include the time taken to generate the set of alignments.

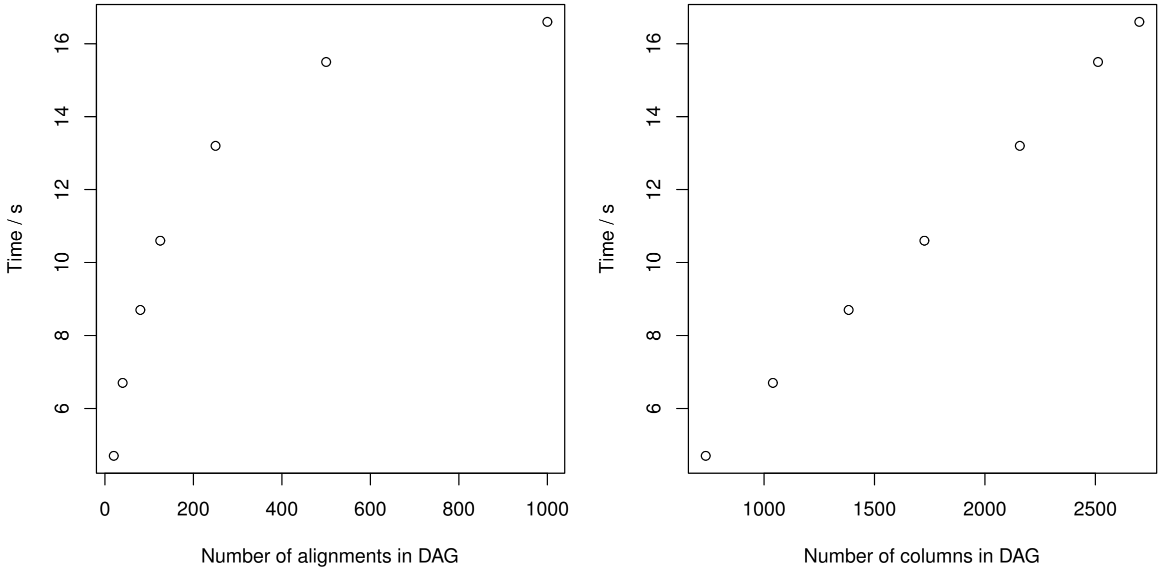

Figure S10 Total runtime for marginal likelihood computations for 2000 trees for the 4-globin example discussed in the main text, versus the number of alignments in the DAG (left), and the total number of columns (right), showing the expected linear scaling.
